# An integrative review of nonobvious puzzles of cellular and molecular cardiooncology

**DOI:** 10.1186/s11658-023-00451-y

**Published:** 2023-05-23

**Authors:** Paweł Uruski, Julia Matuszewska, Aleksandra Leśniewska, Daniel Rychlewski, Arkadiusz Niklas, Justyna Mikuła-Pietrasik, Andrzej Tykarski, Krzysztof Książek

**Affiliations:** 1 Department of Hypertensiology, Poznań University of Medical Sciences, Długa ½ Str., 61-848 Poznan, Poland; 2 Department of Pathophysiology of Ageing and Civilization Diseases, Poznań University of Medical Sciences, Długa ½ Str., 61-848 Poznan, Poland

**Keywords:** Cardiooncology, Cardiac cells, Cardiotoxicity, Vascular cells

## Abstract

Oncologic patients are subjected to four major treatment types: surgery, radiotherapy, chemotherapy, and immunotherapy. All nonsurgical forms of cancer management are known to potentially violate the structural and functional integrity of the cardiovascular system. The prevalence and severity of cardiotoxicity and vascular abnormalities led to the emergence of a clinical subdiscipline, called cardiooncology. This relatively new, but rapidly expanding area of knowledge, primarily focuses on clinical observations linking the adverse effects of cancer therapy with deteriorated quality of life of cancer survivors and their increased morbidity and mortality. Cellular and molecular determinants of these relations are far less understood, mainly because of several unsolved paths and contradicting findings in the literature. In this article, we provide a comprehensive view of the cellular and molecular etiology of cardiooncology. We pay particular attention to various intracellular processes that arise in cardiomyocytes, vascular endothelial cells, and smooth muscle cells treated in experimentally-controlled conditions in vitro and in vivo with ionizing radiation and drugs representing diverse modes of anti-cancer activity.

## Introduction, or a few words about cardiooncology

The improved and still expanding diagnostic capabilities translating to earlier detection, along with the steadily increasing effectiveness of therapeutic approaches, luckily contribute to the growing population of people who successfully deal with cancer [1]. The therapy, often very forcible and life-threatening, leaves its imprint in cancer survivors in the form of temporary side-effects and/or long-term sustained abnormalities in many organs and tissues [2, 3]. One of the most affected systems by therapy is the cardiovascular system (CVS) [4]. Adult patients who survived cancer are more prone to develop cardiovascular disease (CVD) than representatives of the general population [5]. Indeed, CVD belongs to the leading causes of morbidity and mortality in cancer survivors [6–8]. Cardio- and vasotoxicity in oncology patients is so widespread that a new clinical subdiscipline, cardiooncology, has dynamically emerged [4, 9].

At the moment, cardiooncology is a rapidly developing area of science, but purely clinical observations primarily drive this development. However, the pathological phenomena seen in the CVS in patients with cancer have their basis in pathophysiological processes occurring at the cellular and molecular levels. In our opinion, this aspect of cardiooncology is not sufficiently harmonized on a cause-and-effect basis with clinical observations, resulting in an excessive separation of clinical observations from basic research. Therefore, in this article we confront cardiooncology observed from a patient-oriented perspective, with results of experimental studies that allow gathering the causes and effects of specific dysfunctions into a coherent whole.

### Cardiovascular complications in patients with cancer

The CVS disorders that occur in oncology patients undergoing radio- and chemotherapy are driven by a broad range of morphological and functional disturbances within the heart muscle and blood vessels, generally leading to heart failure (HF). Radiation-induced heart disease (RIHD) is the most frequent malfunction that develops in patients experiencing ionizing radiation (IR). The disorder is manifested by a wide range of clinical symptoms, including pericarditis, coronary artery disease, myocardial defects, and inappropriate conduction [10]. Cardiac fibrosis is the most prominent structural defect that is a consequence of both irradiation and chemotherapy, leading to arrhythmia, usually several years after treatment [11]. The functional abnormalities within CVS are far more extensive. They include myocardial dysfunction, myocardial ischemia, valvular heart disease, pericarditis, defective conduction, and arrhythmias. In addition, patients may suffer from some perturbations within systemic and pulmonary blood vessels, including thromboembolic disease, and pulmonary and arterial hypertension. Some of the treatments may also cause more complex perturbations with a strong metabolic component in a long-term perspective, such as: obesity, hyperlipidemia, atherosclerosis, and diabetes [9, 12, 13]. Importantly, therapy-induced cardiotoxicity gives the adverse symptoms that are not limited to resting CVS parameters (e.g., left ventricular ejection fraction). They also engage dynamic cardiac functions, whose deterioration jeopardizes daily quality of cancer survivors’ life, i.a. by interfering with the peak (VO_2peak_) oxygen consumption and translating to reduced exercise tolerance [14].

The incidence of iatrogenic complications is randomly distributed, depending on the treatment regimen and patient-specific conditions. Their frequency ranges from a few percent to tens of percent of individuals [13]. Cardiologic side-effects of cancer therapy exist in patients with various malignancies, treated with IR and drugs representing multiple groups and modes of action. The most recognized in this area are anthracyclines, platins, taxanes, antimetabolites, proteasome inhibitors, anti-HER2 agents, and immune checkpoint inhibitors [13, 15]. Basic characteristics of radiotherapy and drugs affecting CVS and the most commonly observed therapy-related complications are collected in Table 1.

**Table 1:** Functional and clinical characterization of drugs affecting CVS system

Mechanism(s) of anti-cancer activity	Group	Drug	Primary use	CVS disorder/dysfunction
Inhibition of DNA and RNA synthesis by intercalation between base pairs, inhibition of topoisomerase II, an impairment of DNA repair, ROS-induced DNA damage [13]	Anthracyclines	Doxorubicin	ALL, bladder cancer, breast cancer, Ewing sarcoma, endometrial carcinoma, lymphoma, multiple myeloma, hepatocellular cancer, HL, NHL, SCLC, Waldenström macroglobulinemia, osteosarcoma, thymoma. soft-tissue sarcoma, uterine sarcoma [15]	HF, cardiomyopathy, arrhythmias, pericarditis, myocarditis, ventricular arrhythmias, sinus tachycardia, heart block, ECG changes (nonspecific ST and T, decreased QRS voltage, prolongation of QT interval), palpitations [15, 251, 501]
		Daunorubicin	AML, ALL [502]	ECG changes (nonspecific ST and T, decreased QRS, prolongation of QT interval), LVF, myocarditis [251, 503]
		Epirubicin	Gastric cancer, breast cancer, esophageal cancer, osteosarcoma, soft-tissue sarcoma [15]	HF [251, 504]
		Idarubicin	AML [15]	HF, arrhythmias, angina, myocardial infarction [13, 15, 251]
DNA damage (cross-linking of DNA strands, impaired base pairing, strand breaks) [505]	Alkylating agents	Busulfan	Essential thrombocythemia, polycythemia vera [15]	HF, palpitations, cardiac tamponade, cardiomegaly, pericardial effusion, hypokinesis of the ventricular apex, ECG changes (flat T waves or peaked P waves) [251]
		Cyclophosphamide	ALL, breast cancer, CLL, Ewing sarcoma, HL, NHL, multiple myeloma, SCLC [15]	Arrhythmias, chest pain, pleural and pericardial effusions, myocarditis, pericarditis, myocardial infarction, cardiac tamponade, HF, ECG changes (loss of QRS voltage) [251, 503, 506]
		Ifosfamide	Bladder cancer, cervical cancer, Ewing sarcoma, HL, NHL, osteosarcoma, soft-tissue sarcoma, testicular cancer, thymoma, ovarian cancer [15]	Pleural and pericardial effusions, reentrant ventricular tachycardia, pulseless tachycardia, ECG changes (nonspecific ST and T, loss of QRS voltage) [251]
		Melphalan	Multiple myeloma, ovarian cancer, HL, lymphomas [15]	Arrhythmias, atrial fibrillation [15]
Inhibition of DNA replication due to depletion of nucleotides, induction of DNA and RNA strand breaks, induction of apoptosis [13]	Antimetabolites	5-Fluorouracil	Bladder cancer, breast cancer, cervical cancer, colorectal cancer, esophageal cancer, gastric cancer, hepatobiliary cancer, pancreatic cancer, squamous cell carcinomas [15]	Angina, chest pain, ECG changes (ST-segment changes, T wave abnormalities), palpitation, dyspnea, coronary vasospasm, tachycardia, bradycardia, hypotension, HF, myocardial ischemia [13, 251, 507]
		Capecitabine	Breast cancer, colorectal cancer, gastric cancer, hepatobiliary cancer, esophageal cancer, ovarian cancer, fallopian peritoneal cancer, pancreatic cancer [15]	Chest pain, palpitation, dyspnea, hypotension, tachycardia, bradycardia, hypertension, HF, myocardial ischemia [507, 508]
		Gemcitabine	Adenocarcinoma, breast cancer, bladder cancer, cervical cancer, head and neck cancer, hepatobiliary cancer, HL, NHL, malignant pleural mesothelioma, NHL, NSCLC, ovarian cancer, pancreatic cancer, sarcoma, SCLC, testicular cancer, uterine cancer [15]	Coronary vasospasm, capillary leak syndrome, arrhythmias, myocardial ischemia and infarction, LVEF reduction [503, 509, 510]
Hyperstabilization of microtubules [13, 511]	Taxanes	Paclitaxel	Adenocarcinoma, bladder cancer, breast cancer, cervical cancer, head and neck cancers, Kaposi sarcoma, NSCLC, esophageal and gastric cancer, ovarian cancer, SCLC, soft-tissue sarcoma, testicular germ cell tumor, thymoma [15]	Atrial and ventricular arrhythmias, atrial fibrillation, atrial flutter, atrial tachycardias, sinus bradycardia left bundle branch block, myocardial ischemia [13, 15, 251]
		Docetaxel	Adenocarcinoma, bladder cancer, breast cancer, Ewing sarcoma, head and neck cancer, NSCLC, esophageal and gastric cancer, ovarian cancer, prostate cancer, SCLC, soft-tissue sarcoma [15]	Arrhythmias, atrial fibrillation, atrial flutter, atrial tachycardias, bradycardia, ECG changes (QT prolongation) [13, 501, 503]
Inhibition of microtubule formation in mitotic spindle [512, 513]	Alkaloids	Vinblastine	Bladder cancer, HL, melanoma, NSCLC, soft-tissue sarcoma, testicular cancer [15]	Myocardial ischemia [514]
		Vincristine	ALL, HL, NHL, Ewing sarcoma, gestational trophoblastic tumor, multiple myeloma, ovarian cancer, primary CNS lymphoma, SCLC, thymoma [15]	Myocardial infraction; myocardial ischemia, ECG changes (T wave inversions, ST segment depression), atrial fibrillation, LVF [251, 503]
Binding and cross-linking within DNA, preventing its replication, induction of apoptosis [515]	Platins	Cisplatin	Bladder cancer, breast cancer, cervical cancer, endometrial carcinoma, esophageal and gastric cancer, head and neck cancer, HL, malignant pleural mesothelioma, multiple myeloma, NHL, osteosarcoma, ovarian cancer, penile cancer, SCLC, testicular cancer [15]	HF, arrhythmias, interventricular block myocardial infraction, hypotension, ECG changes (ST-T wave, T wave inversion) [251]
		Oxaliplatin	Biliary adenocarcinoma, CLL, colorectal cancer, neuroendocrine tumor (carcinoid), NHL, ovarian cancer, esophageal/gastric cancer, pancreatic cancer, testicular cancer [15]	Arrhythmias [15]
Induction of DNA strand breaks [516, 517]	Antitumor antibiotics	Bleomycin	HL, testicular cancer, ovarian germ cell cancer [15]	Myopericarditis, myocardial ischemia [503, 518]
Induction of apoptosis, inhibition of angiogenesis [519]	Immunomodulatory drugs	Lenalidomide	CLL, diffuse large B cell lymphoma, MCL, multiple myeloma, MDS [15]	Arterial thromboembolism disease, myocardial infraction, cerebral vascular events, deep vein thrombosis, pulmonary embolism [520]
Inhibition of HER2 tyrosine kinase [13]	Humanized monoclonal antibodies	Trastuzumab	Breast cancer, gastric cancer [15]	HF, myocardial necrosis/fibrosis, LVEF decline, HF, LFV [503, 521]
Inhibition of VEGFR kinases [522]	Multi-target kinase inhibitors	Lenvatinib	Hepatocellular cancer, renal cell carcinoma, thyroid cancer [15]	Hypertension, HF, ECG changes (QT interval prolongation) [523, 524]
		Pazopanib	Renal cell carcinoma, soft-tissue carcinoma, thyroid cancer [15]	HF, LVEF reduction [525]
		Vandetanib	Thyroid cancer [15]	HF, tachycardia, palpitation, ECG changes (QT prolongation), LVEF reduction [524]
Inhibition of proteasomal protein degradation leading to apoptosis and ER stress [526]	Proteasome inhibitors	Bortezomib	Follicular lymphoma, mantle cell lymphoma, multiple myeloma, T-cell lymphoma [15]	HF, coronary heart disease, arrhythmias, thromboembolic disease, pericardial complications, peripheral vascular disease [527]
		Carfilzomib	Multiple myeloma, Waldenström macroglobulinemia [15]	HF, arrhythmias, hypertension, myocardial ischemia [382, 503]
Reactivation of immunosurveillance mechanisms, particularly T-cell activity [455]	Immune checkpoint inhibitors	Ipilimumab (anti-CTLA-4)	Colorectal cancer, melanoma, renal cell carcinoma [455]	Arrythmias, pericarditis, myocarditis [15, 528]
		Nivolumab (anti-PD-1)	Colorectal cancer, hepatocellular carcinoma, HL, melanoma, NSCLC, SCLC, urothelial carcinoma [15]	Arrythmias, peripheral edema, hypertension [15]
		Pembrolizumab (anti-PD-1)	Cervical cancer, gastric cancer, hepatocellular carcinoma; HL, melanoma, Merkel cell carcinoma, NSCLC, urothelial carcinoma [15]	Arrythmias, HF, edema, pericarditis, pericardial effusion [15, 528]
		Atezolizumab (anti-PD-L1)	Breast cancer (triple negative), NSCLC, SCLC, urothelial carcinoma [15]	Myocardial infarction [528]

*AML* acute myeloid leukemia, *ALL* acute lymphocytic leukemia, *CLL* chronic lymphocytic leukemia, *APML* acute promyelocytic leukemia, *HL* Hodgkin lymphoma, *NHL* non-Hodgkin lymphoma, *MCL* Mantle cell lymphoma, *MDS* myelodysplastic syndromes, *CNS* central nervous system, *SCLC* small cell lung carcinoma, *NSCLC* non-small cell lung cancer, *HF* heart failure, *LVF* left ventricular failure, *LVEF* left ventricular ejection fraction

Mechanistically, the cardiologic disorders that develop in cancer survivors have cellular and molecular determinants. The most important include cellular energetics, electrophysiology, growth and differentiation, proteostasis, senescence, regulation of gene expression (methylation, microRNAs), DNA damage, oxidative stress, inflammation, and cell death (apoptosis, ferroptosis, autophagy, mitophagy). Various mutually interacting signaling pathways control these events at the upstream and downstream levels, and cellular transcriptome and proteome alterations trigger their changes. Because the above-mentioned effectory mechanisms of CVS dysfunction have their specific topography within the heart and blood vessels, their appearance upon radiation and drug exposure will be described and discussed from the perspective of three major cell types forming these multicellular organs and working in concert for the right heart and vessel activity. These will be cardiomyocytes, comprising ~ 80% of the cellular heart volume and responsible for its electro-mechanical activity [16], and endothelial cells and smooth muscle cells that interact, creating heart vascularization and controlling peripheral blood vessel organization and function [17].

### Mechanisms of IR-dependent cardiotoxicity and vasotoxicity

**Fig. 1:**
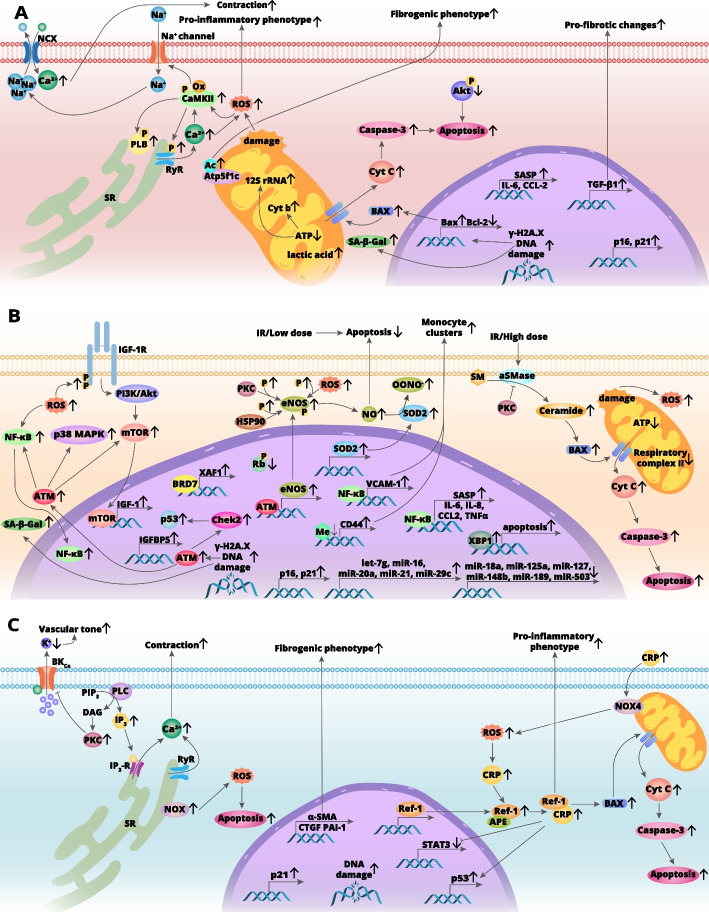
Pathomechanisms of IR-dependent cardio- and vasotoxicity in cardiomyocytes (**A**), vascular endothelial cells (**B**), and vascular smooth muscle cells (**C**). *AC* acetylation, *Me* methylation, *SM* sphingomyelin, *OONO*^*−*^ peroxynitrite, *aSMase* acid sphingomyelinase, *PIP*_*3*_ phosphatidylinositol (3,4,5)-trisphosphate, *PLC* phospholipase C, *DAG* diacylglycerol, *IP*_*3*_ inositol 1,4,5-trisphosphate, *IP*_*3*_*-R* inositol 1,4,5-trisphosphate receptor

Currently, radiotherapy employing gamma and X-rays is applied for about half of all malignancies, and for some, it is the primary treatment modality [18, 19]. At the same time, the molecular background of RIHD that may occur from months to decades following exposure to either oncologically effective doses of IR or low doses of radiation resulting from occupational contacts [20–22] is still not sufficiently understood (Fig. 1).

#### Cardiomyocytes

Considering the role of cardiac muscle cells in electrophysiological heart activity, it is more than plausible that IR seriously violates their biology. Experiments on human-induced pluripotent stem cell-derived cardiomyocytes (hiPSC-CMs), a model resembling primary cardiomyocytes [23], allowed for the determination of the radiation doses (5 and 10 Gy) and exposure times [120], reducing cell viability. The cells remained alive when the treatment lasted shorter (e.g., 48 h) or the dose was lower (< 5 Gy). The irradiation performed within dose and time frames, ensuring unaltered viability, revealed that the spontaneous pacemaker activity of the cells was also unaffected. This situation has changed and the beating frequency declined when the hiPSC-CMs were subjected to higher doses of radiation for at least 48 h [24]. Interestingly, cardiomyocyte response to IR was not uniform, because some cells seemed more sensitive to the stressor [24]. This effect was probably associated with the spatial distribution of the cells subjected to IR and their contribution to the formation of the pacemaker center. This random responsiveness of cardiomyocytes to radiation could explain, at least partly, a broad and nonspecific population distribution of arrhythmia [25] and its relatively low frequency (~ 4–5%) among other CVS disorders in IR-treated patients with cancer [26]. Another issue that has to be solved is whether IR doses identified as reducing cardiomyocyte viability translate to in vivo conditions, e.g., in the context of tolerance doses (40 Gy) [27]. However, a trend regarding irradiation doses with potentially cardiotoxic outcomes is recently decreasing and points to the value of 15 Gy as the threshold [28].

The heart muscle loses contractile cells during a lifetime and in response to various challenges. At the same time, the organ has minimal capacity to regenerate because of the postmitotic state of most adult cardiomyocytes [29]. Some hopes regarding improving heart regeneration are associated with the cardiogenic reprogramming of pluripotent stem cells [30]. The radiation issue is thus of particular importance, considering the potential risk of tumorigenesis after stem cell implantation. Unfortunately, the data on the sensitivity of differentiated hiPSC-CMs and their pluripotent precursors are inconclusive. For example, the pluripotent cells giving rise to hiPSC-CMs are more resistant to IR (utilized at cGy doses) than the differentiated cells, as their viability and proliferation were unchanged conversely to the insufficient number of functional, autonomously beating cells [31]. Other authors, also working with irradiated hiPSC-CMs demonstrated that their proliferative capacity does not differ from that characterizing untreated cells, and the same applies to the incidence of apoptosis. At the same time, they postulate that irradiation eliminates highly proliferative pluripotent stem cells that are more sensitive to this stressor. To strengthen this thesis, they employed NOG mice and showed that tumor formation was less frequent in the irradiated than in the unirradiated group, which could be associated with IR-dependent reduction of the number of undifferentiated hiPSC [32], likely due to induction of their apoptotic death [33]. One may not exclude that these discrepancies in IR sensitivity of immature stem cells and differentiated cardiomyocytes may result from experimentally tested doses of radiation. The report showing unaltered proliferation and apoptosis of hiPSC-CMs with a concomitant deterioration of stem cells is based on markedly higher IR dosage (2–8 Gy) than the study demonstrating the opposite findings [31, 32].

Regarding the electrophysiological activity of cardiomyocytes, the irradiated cells display increased predilection to spontaneous release of Ca^2+^ upon the β-adrenergic stimulation by isoproterenol. This effect may explain, at least to some extent, the proarrhythmic tendency of the heart under adrenergic stress [34]. Further dedicated studies in this respect are, however, needed because the intensity of IR-related Ca^2+^ waves in cardiomyocytes may depend on the time of exposure. The amplitude of Ca^2+^ transient increases in the short-term perspective, whereas the prolonged trend (1 week) decreases, corresponding with reduced left ventricular contractility in anesthetized mice. This effect may be probably related to chronically reduced sarcoplasmic reticulum (SR) calcium content [35]. Another issue is that the contractile activity of IR-treated cardiomyocytes is determined not only by Ca^2+^ level. The cells responded dose-dependently to IR, also by increasing Na^+^ content, which resulted in Na^+^-dependent Ca^2+^ overloading through the Na^+^/Ca^2+^ exchanger (NCX) [35].

Adrenergic stimulation of the heart leads to Ca^2+^ overload and oxidative stress [36]. The role of oxidative stress, understood as the disproportion between the generation and removal of reactive oxygen species (ROS) leading to macromolecular damage [37], in CVS dysfunction is indisputable [38]. Discoveries convincingly connect this phenomenon with cardiovascular challenges seen in oncologic patients undergoing various forms of therapy [39]. Although the list of oxidative stress culprits is long, IR is among the most prominent external causes [40]. A hit of ^137^Cs *γ* rays induces the production of ~ 60 ROS/ng of tissue in less than one microsecond [41]. When cardiomyocytes are exposed to even low doses of IR (10 cGy) for 12 days, they exhibit an upregulated expression of transcripts for mitochondrially encoded cytochrome b and 12S ribosomal RNA. Because these transcripts are engaged in electron transport [42] and mitochondrial protein biogenesis [43], the above finding implies that IR intensifies mitochondrial metabolism. These changes may also exemplify a compensatory cell response to restore ATP content that could deteriorate due to IR-related mitochondrial oxidative phosphorylation system failure [31, 44]. The theory of IR-dependent intensification of cellular metabolism followed by ATP depletion was proved by another group of scientists who found an increased expression of some energetic proteins, like fatty acid synthase and solute carrier family 25 member 1 in irradiated mouse hearts. At the same time, the tissues displayed injured mitochondrial inner membranes, decreased ATP content, and accumulated lactic acid [45]. Changes in the cardiomyocyte mitochondrial structure belong, in fact, to the oldest observation of IR-related cellular damage. In 1968, it was described that high doses of radiation (52 Gy) seriously affect the mitochondria architecture, exemplified by their swelling, abnormalities within the cristae, and bonded outer double membranes [46].

Following the trait of increased mitochondrial metabolism, one may anticipate that the production of ROS, treated as by-products of respiratory chain reactions, should also be elevated [47]. This general assumption agrees with experiments using isolated ventricular mouse cardiomyocytes, which overproduced ROS upon irradiation [35]. These elevated ROS could be another factor responsible for altered cardiac Ca^2+^ handling, particularly its efflux from SR [48]. This statement is based on the observation that electrophysiological effects of IR-dependent oxidative stress (e.g., the diminished systolic Ca^2+^ transients) were reversible by antioxidants. IR-dependent oxidative stress in cardiomyocytes was mediated by Ca^2+^/calmodulin-dependent protein kinase II (CaMKII). The activity of this kinase was persistently upregulated after irradiation and correlated with hyperphosphorylation of its targets, RyR2 and PLB [35].

ROS-dependent changes in the mechanical action of the heart are not the only consequences of IR. Decreased viability of irradiated cardiomyocytes may be underlined by excessive DNA injury, leading to apoptosis. Such a sequence of events was observed, e.g., in rat embryonic ventricular H9C2 cardiomyocytes, whose treatment with IR (at 2, 5, 10, and 15 Gy) resulted in a dose-dependent increase in the percentage of apoptotic cells. An upregulated level of proapoptotic protein Bax and a downregulated level of anti-apoptotic protein Bcl-2 accompanied this effect. The augmentation of apoptosis coincided with a dose-dependent induction of ROS and accumulation of DNA damage foci (γ-H2A.X) [49]. The vulnerability of the tested cells to IR may also result from decreased phosphorylation of Akt [49], a multiplayer kinase involved e.g., in the maintenance of the respiratory status of mitochondria, decreasing ROS efflux, and cell self-repair [50]. The activity of PI3K/Akt pathway, cooperating with HER2 signaling, has also been found to be essential for controlling the appropriate functioning of the heart, including the survival of cardiomyocytes [51].

However, IR-dependent apoptosis of cardiomyocytes should be treated with caution because its induction, also reported by other groups [52], seems to not be a universal phenomenon. Moreover, although an increased frequency of apoptosis was found under in vitro cell culture conditions, this outcome was not confirmed by the same group in vivo [49], which may indicate that the cellular model is more sensitive to radiation than tissue structures. Moreover, in the in vivo conditions, the irradiated cardiomyocytes may die due to direct exposure to IR and indirectly due to a reduced vessel density contributing to the formation of ischemic areas [52]. In turn, cardiomyocytes that survived the direct and indirect radiation became hypertrophic to compensate for previous cell loss [52]. Another consequence of this heart remodeling is the accumulation of fibrous tissue, leading to the next and the most critical morphological sign of RIHD, fibrosis [53]. The profibrotic changes observed in the irradiated hearts may be partly associated with the overproduction of transforming growth factor-β1 (TGF-β1) [52, 54], which is one of the key mediators of extracellular matrix (ECM) protein (e.g., collagen) deposition and the loss of tissue elasticity [55].

The phosphorylated variant of histone H2A.X, the γ-H2A.X, is the marker of DNA double-strand breaks involved in the DNA damage response (DDR) reaction. The accumulation of γ-H2A.X foci assembling to injured DNA is a sign of cellular senescence [56]. This phenomenon represents an irreversible cell growth cessation caused by excessive and irreparable DNA damage [57]. Senescent cells accumulate in vivo with age and participate in several pathological processes like chronic inflammation, tissue fibrosis, and cancer [58]. The harmful activity of senescent cells proceeds mainly via their ability to hypersecrete various proinflammatory and ECM remodeling proteins to the environment, known as the senescence-associated secretory phenotype (SASP) [59]. The observation of the γ-H2A.X induction in the irradiated H9C2 cardiomyocytes [49] was positively verified and additionally strengthened by the demonstration of the presence of other senescence indices in these cells. These included an elevated expression of senescence-associated β-galactosidase (SA-β-Gal), upregulated levels of cell cycle inhibitory proteins p16 and p21, and increased expression of transcripts coding for some SASP factors, like IL-6, CCL-2, MMP-2, Col3a1, and CTGF in the irradiated H9C2 cells. Significantly, these cellular changes corresponded to the formation of senescence phenotype in the heart tissue in vivo, in which IR treatment resulted in the telomere shortening and even more excessive SASP formation [60].

IR-induced cardiomyocyte senescence may be associated with lysine acetylation, one of the most critical post-translational protein modifications [61]. Previously, transient lysine acetylation was found to play a regulatory role in cardiological abnormalities like arrhythmia, HF, and hypertension [62]. The hearts of irradiated animals and irradiated cardiomyocytes displayed upregulated lysine residue acetylation level. Nearly half of the hyperacetylated proteins were localized within the mitochondria. Differentially expressed proteins in the acetylation sites were grouped mainly in energy production and conversion, fatty acid and amino acid transport, and utilization phenomena. One of the hyperacetylated targets, Atp5f1c (the lysine 55 site), which is closely related to cell energetics as an element of an ATP synthase complex [63], appeared to be causatively connected with the development of IR-driven senescence phenotype in H9C2 cells. Hypothetically, it could also contribute to fibrotic changes in the heart muscle in vivo [60].

#### Endothelial cells

Vascular dysfunction is one of the most common complications of radiotherapy [64]. Retrospective, histopathological examination of patients with rectal cancer subjected to preoperative irradiations revealed numerous signs of vasotoxicity, including vascular dystrophy and hypertrophy, vascular and perivascular fibrosis, and intimal hyperplasia connected with luminal narrowing. The vessels were also characterized by increased collagen I and III content [65]. Echocardiographic analysis of irradiated mouse hearts showed a diminished intramyocardial vessel-to-cardiomyocyte ratio in the left ventricle, which is suggestive of retrograde vascular lesions within the cardiac muscle [52]. Irradiated pig coronary arteries revealed, in turn, the presence of perivascular adhesions, vessel enlargement, a thickened adventitia, adventitial neovascularization, and medial necrosis [66]. The damage that develops within the cardiomyocytes in response to IR (discussed in the previous section) is also associated with cell ischemia [67] triggered by injured cardiac nutrient-supplying vessels, i.a. due to apoptosis of endothelial cells [68].

Considering the prominent role of endothelial cells (ECs) in vascular functioning, the above-mentioned morphological disturbances within blood vessels are most likely determined by certain disturbances in EC structure and function. As per the former, scanning electron microscopy revealed that ECs in the irradiated arteries form an intact layer of cells that display a hypertrophic appearance [66]. One of the possible reasons for cellular hypertrophy, usually caused by impaired proteostasis [69], is cellular senescence [70]. Indeed, ECs display several features of senescence phenotype after irradiation with moderate-to-high doses of radiation in vitro [71–73]. Irradiated (4 Gy) human umbilical vein endothelial cells (HUVECs) are characterized by a hypertrophic and flattened shape, depressed proliferation, and increased expression of SA-β-Gal, p16, and p21 proteins. Moreover, they display decreased phosphorylation of retinoblastoma protein (pRb) and the activation of DDR, represented by accumulated γ-H2A.X foci and activated protein kinase ataxia telangiectasia mutated (ATM), Chek2, and p53 [72]. A similar EC phenotype was also seen after the in vivo irradiation of experimental animals [74, 75]. A simultaneous upregulation of p16 and p21 in the irradiated, senescent ECs [72, 76] implies that both telomere-dependent [77] and telomere-independent [78] types of senescence machinery are involved. However, the activation of DDR is not required to maintain a senescent state, as rat cerebromicrovascular ECs remained senescent despite near total repair of injured DNA [79]. In turn, the signaling involving activated p53 appeared to be critical for IR-dependent senescence of human lung microvascular ECs (HLMVECs) [76].

The remaining effectory pathways associated with IR-dependent EC senescence are numerous and seem to define the type of investigated cells. Irradiated senescent HUVECs exhibit increased expression of mRNA and production of protein for insulin-like growth factor binding protein 5 (IGFBP5), recognized in the knockdown interventions as the contributing agent in their diminished proliferation, activated SA-β-Gal, and impaired tube formation ability [72]. In pulmonary microvascular endothelium, which displays the morphology as the IR-treated HUVECs, the senescence response that approached after their irradiation was elicited by an X-linked inhibitor of apoptosis-associated factor 1 (XAF1).

The XAF1 is transcriptionally regulated by bromodomain 7 (BRD7), the expression of which increases at both mRNA and protein levels in response to IR, and the downregulation of which leads to a partial reversal of senescence [80]. Studies on human pulmonary artery ECs (HPAEC) showed, in turn, that IR applied at the dose of 10 Gy induced their premature senescence in a mechanism involving a phosphatidylinositol-3-kinase (PI3K) and mammalian target of rapamycin (mTOR) signaling. The involvement of the mTOR pathway was associated with the overproduction of insulin-like growth factor-1 (IGF-1) and hyperphosphorylation of IGF-1 receptor (IGF-1R), the activity of which is upstream of mTOR [73].

Functionally, senescent HUVECs display decreased angiogenic reactions, manifested by deteriorated ability to form capillaries on Matrigel™. This feature was surprising, considering their increased migratory potential, causatively linked with overexpressed matrix metalloproteinase 2 (MMP-2) [72]. The issue of IR-dependent changes in EC motility needs to be further explored, also in the context of findings by other groups who observed that HUVEC irradiation using doses up to 8 Gy led to a depression of their migration, invasion, and the ability to form capillaries [71].

Functional perturbations associated with IR-related toxicity and/or EC senescence are primarily related to the pathogenesis of atherosclerosis. A pathomorphological examination of atherosclerotic arteries indicated the presence of senescent cells in vivo, whereas eliminating these cells decelerated the disease progression [81–83]. In this context, the ambiguous role of nitric oxide (NO), the central smooth muscle relaxant [84], is especially intriguing. Classically, senescent ECs are characterized by decreased activity of endothelial nitric oxide synthase (eNOS), leading to NO’s declined generation [85, 86]. Experimental data suggest that NO deficits may causatively participate in EC senescence, as the augmentation of this molecule level appears to inhibit the process. Because this effect coincided with the delayed senescence-associated deterioration of telomerase activity, one may assume that the antisenescence activity of NO was associated with the restoration of telomeric DNA [87]. Analogical findings were derived from the study in which HUVEC transfection with eNOS or their treatment with NO donor reduced the fraction of SA-β-Gal-positive cells. The antisenescence activity of NO was attributed to its antioxidative properties, particularly the ability to neutralize ROS overproduced during EC senescence [81]. The perception of NO function in EC senescence changes radically when IR induces the process. In such conditions, the eNOS-NO axis seems integral to radiation-induced DDR and to the “bad guy” during EC senescence. It has been found that IR induced the eNOS and NO dose-dependently, leading to increased cell migration and sprouting, which corresponded with increased tumor angiogenesis in vivo [88]. IR-treated bovine aortic EC displayed increased eNOS transcription and NO production, and these effects were regulated by upregulated DNA damage-sensing ATM [89]. The same kinase along with heat shock protein 90 (HSP90), also operating within the DDR [90], was also found to be responsible for the phosphorylation of serine 1179 of eNOS, another important step of the enzyme activation [91]. The HUVEC irradiation (< 20 Gy) also led to increased eNOS phosphorylation at Ser-1177 and dephosphorylation at Thr-495, and these events were mediated by increased activity of protein kinase C-βII (PKC-βII) [92]. As a consequence of radiation-induced NO, the cells exhibited decreased cell death incidence and intensified senescence [89]. At the same time, their exposure to N-nitro-l-arginine methyl ester (l-NAME), the NO inhibitor, prevented senescence development after the IR challenge [93]. As per the possible sequence of events staying behind the IR-induced eNOS activity and NO release, activated ATM translocates to the cytosol to stimulate its down-stream effectors, such as NF-κB [94], mTORC_1_ [95], and p38 MAPK [96]. Therefore, this is the right place to conclude that although NO overproduction, in general, is regarded as a favorable phenomenon from the point of view of CVS function, its excess resulting from cell irradiation should instead be considered as an adverse effect. This prediction was confirmed in several animal models in which uncoupling of NOS and exacerbated mitochondrial ROS generation led to dilatory remodeling and cardiac dysfunction [97]. And conversely, the loss of inducible NOS (iNOS) diminished cardiac remodeling and improved cardiac reserve, postmyocardial infarction [98]. One cannot exclude that these adverse outcomes of NO activity may occur because this molecule may subtly react with mitochondrial superoxides leading to the formation of the highly active and deleterious peroxynitrite [99].

Another aspect of the IR-dependent contribution of senescent EC to atherosclerosis, particularly at its early stage, is monocyte recruitment into the blood vessel wall [100]. The presence of hypertrophic endothelium within atherosclerotic lesions has a long history dating back to when this phenomenon was not linked with senescence phenotype [101, 102]. Human coronary artery ECs irradiated with 10 Gy adopted an enlarged SA-β-Gal-positive appearance and displayed an increased capacity to bind monocytes. The adhesion-promoting activity of senescent ECs was associated with upregulated CD44, allowing them to form monocyte clusters on the surface of senescent cells. This receptor’s role in atherosclerosis pathogenesis was previously established in vivo as the incidence of atherosclerotic lesions in CD44 null animals and was markedly reduced [103]. Increased expression of CD44 in senescent ECs, which another group also reported in irradiated senescent HUVECs [72], was in turn regulated by radiation-dependent demethylation of its promoter CpGs at positions −638 (CpG1), −627 (CpG2), and −607 (CpG3). Of them, the CpG3, whose hypomethylation was the greatest [100], has previously been linked with the activation of the CD44 promoter [104]. It is worth noting that DNA methylation is a potent epigenetic regulator responsible for transcriptional gene silencing. An overall decline in DNA methylation is a well-established feature of senescent cells, contributing to decreased transcription of either immune response genes (CpG sites) or SASP-related genes (non-CpG islands) [105]. The concept of epigenetic regulation of increased monocyte adhesion to senescent ECs is in line with other studies of the same phenomenon, in which the pro-adhesive capacity of cells was linked with an overproduced VCAM-1. Significantly, the VCAM-1 gene promoter was characterized by the open chromatin state due to increased methylation of histone H3 on lysine 4 (H3K4me3), which allowed for its increased accessibility and the transcriptional activity of NF-κB [106].

Another regulatory role regarding gene expression plays short, non-coding RNA molecules (microRNAs/miRNAs) that generally affect this process as its repressors [107]. Experimental overexpression of some miRNAs were found to ameliorate oxidative stress in cardiac cells, serving as potential therapeutic targets in the course of cardiomyopathy [108]. It was demonstrated that IR (2 Gy) modulates miRNA levels in human dermal microvascular endothelial cells (HDMECs). The upregulated targets included let-7 g, miR-16, miR-20a, miR-21, and miR-29c, whereas the expression of miR-18a, miR-125a, miR-127, miR-148b, miR-189, and miR-503 was decreased. The observed changes were biologically valid, as those regarding let-7 g, miR-189, and miR-20a corresponded with cell survival and proliferation, those associated with miR-125a and miR-189 were responsible for radioprotection, whereas those linked with miR-127 and let-7 g contributed to increased radiosensitivity [109].

The pathogenesis of radiation-induced CVS disorders has a strong inflammatory component [110], which is associated to a large extent with cellular senescence potentiating extracellular inflammation due to the development of SASP. For example, microvascular HMVEC-L cells that senesced upon IR exposure displayed increased production of IL-8 [76], whereas HUVECs hyperexpressed mRNA for IL-6 [71]. One of the very first events that underlie inflammation-based cardio- and vasotoxicity is the activation of nuclear factor κB (NF-κB), a molecule prone to be activated in ECs in response to genotoxic action of IR [71, 111] and playing the central role for the SASP development [112]. In vascular endothelium, ROS, DNA injury, and damage-associated molecular patterns (DAMPs) released by injured cells activate NF-κB. Following NF-κB activation and nuclear translocation, the irradiated ECs overproduce and/or hypersecrete an extensive repertoire of proinflammatory agents, including IFNγ, TNFα, TGF-β, IL-1, IL-6, CCL2, CXCL8, ICAM-1, VCAM-1, and E-selectin [113]. Interestingly, the NF-κB-dependent signaling is also crucial for IR-driven EC senescence itself, as the chemical blockade of its essential modulator (NEMO) inhibited the formation of senescence phenotype in the irradiated cells [71].

DAMPs released from breast cancer cells dying after their irradiation (20 Gy) directly promote the recruitment of neutrophils and monocytes in vivo. Significantly, the DAMPs, particularly HSP70, HMGB1, and S100A8/A9, also activate HUVECs through toll-like receptor 4 (TLR4) and/or the TLR2/TLR4 dimer. Upon stimulation, ECs overexpress a variety of surface adhesion molecules (ICAM-1, E-selectin), cytokines, and chemokines (IL-1β, IL-6, CCL2, CXCL1, CXCL8, CXCL2, CCL7) that act as leukocyte chemoattractants [114]. Apart from the activation of NF-κB, DAMPs-related proinflammatory switch also engages other signaling routes, such as mitogen-activated protein kinase (MAPK) and interferon regulatory factor 3 (IRF3) [115, 116]. The irradiation also induces high-mobility group protein-1 (HMGB1) [117], a mediator stimulating human microvascular ECs to produce ICAM-1 and VCAM-1. Moreover, IR-treated ECs display elevated levels of receptors for advanced glycation end products (RAGE), TNFα, PAI-1, and tPA [116].

Another possible cause of a sterile inflammation typical for atherosclerosis is oxidative stress [118], a well-recognized consequence of EC exposure to radiation. Irradiated (15 Gy) microvascular HMVEC-L cells generate increased amounts of mitochondrial ROS, exhibit reduced respiratory complex II activity, and increased superoxide dismutase 2 (SOD2) and glutathione peroxidase 1 (GPX1) expression levels [76]. The causative action of the IR-related oxidative stress confirmed experiments with MnTBAP, the SOD mimetic, the administration of which allowed inhibiting the development of senescence phenotype to a large extent [76]. As per the reasons for mitochondrial dysfunction leading to oxidative stress in irradiated ECs, increased activity of NADPH oxidase plays a role. Brain microvascular ECs subjected to IR undergo oxidative stress accompanied by increased inflammatory indices (NF-κB, ICAM-1), and this phenotype was abolished by pharmacologic and genetic inhibition of the enzyme [119]. NF-κB is, in fact, one of the ROS’s central signaling targets, which exacerbates the inflammation in a self-stimulatory manner [120]. ROS, whose production increased in the irradiated HUVECs, were also engaged in the reported radiation-induced changes in eNOS phosphorylation [92]. It is very plausible that IR-related changes in oxidative EC status largely depend on altered mitochondrial metabolism. Experiments on human telomerase-immortalized coronary artery ECs (TICAE) showed that their irradiation leads to structural disorganization of mitochondria (swelling, cristae disruption), accompanied by lowered basal respiration and ATP synthesis, without changes in mitochondrial oxidative phosphorylation system (OXPHOS) capacity [121]. In contrast to previously cited reports, TICAE irradiation did not result in increased ROS efflux, which may be associated with a relatively low dose of IR (2 Gy) and the fact that telomerase may modulate mitochondria metabolism, counteracting the excessive ROS production [122].

The development of cellular senescence is an alternative cell fate to apoptotic cell death, as the senescent cells are usually resistant to proapoptotic stimuli [123]. As per an interplay between these two processes in irradiated ECs, the final response depends on the strength of the insult. Apoptosis is the primary response to relatively high doses of IR (higher than 10 Gy). It proceeds through the acidic sphingomyelinase-dependent processing of sphingomyelin, leading to the accumulation of ceramide and the upregulation of Bax and Bak proteins [124, 125]. It is hypothesized that the ceramide-dependent mechanism of apoptosis in IR-treated ECs is associated with the exceptionally high activity of this enzyme in comparison with other cell types [126]. At the same time, when irradiation occurs at lower doses, that is between 0.5 and 10 Gy, the development of senescence phenotype is the main reaction [71, 72]. However, this dose-dependency is sometimes obliterated, which may be related to the origin of ECs and the detailed methodological aspects of their experimental irradiation, like culture density and exposure time. Moreover, in some experimental models, like human or bovine pulmonary artery ECs, apoptosis was not detected below a threshold of 30–50 Gy [127]. Above these doses, the apoptosis occurred in a mechanism involving activation of endoplasmic reticulum (ER) stress response and an elevation of the transcription factor X-box binding protein 1 (XBP1) and its downstream target, glucose-regulated protein 78,000 (GRP78), serving as an ER chaperone [127]. Another player in the radiation-dependent apoptosis of ECs is protein kinase C (PKC), which acts as the negative regulator of this process by negating sphingomyelin processing to ceramide [124].

#### Vascular smooth muscle cells

CVS malfunction triggered by IR exposure largely results from changes in vascular smooth muscle cells (SMCs). Regarding CVS disorders’ etiology, vascular SMCs are of particular importance for the development of atherosclerosis [128]. Atherosclerotic plaque SMCs originate from a fraction of mature medial cells that are clonally replicated to settle the lesion [129, 130]. The activity of SMCs that are localized within the plaque depends on the phenotypic transformations of these cells and may exert both positive and negative outcomes for plaque stability [131, 132]. Research on SMCs lineage tracing (*Myh11*-ER^T2^Cre YFP^+^) mice with experimentally-induced atherosclerosis revealed that IR promotes atherosclerotic plaque destabilization in a mechanism involving SMCs [133]. Animal exposure to high doses of radiation (1200 cGy) results in the loss of SMCs’ tendency to colonize atherosclerotic lesions with a concomitant increase in an intraplaque hemorrhage and necrotic core area and decreased collagen content. Mechanistically this effect was linked with the increased extent of DNA damage within SMCs, pointing at the same time to various susceptibilities of these cells to IR, depending on their primary localization. An extensive DNA injury did not translate to an increased incidence of apoptosis, which is in line with another report based on irradiated (1.25–20 Gy) rat aortic SMC [134]. However, increased apoptotic death was evident in human aortic SMCs subjected to IR (4, 8 Gy). It was causatively connected with the upregulation of NADPH oxidase (NOX), followed by a dose-dependent stimulation of ROS release. Another pathway responsible for cell death induction was the upregulation of C-reactive protein (CRP), leading to an increased ROS and apoptosis in IR-treated SMCs [135]. The CRP level correlates with the magnitude of inflammation, and its production increases after irradiation [136]. At the same time, the CRP may mediate SMCs apoptosis through NOX4-dependent overproduction of ROS [137] and induction of caspases [138]. In the case of irradiated SMCs, CRP assembles with AP-endonuclease/Redox factor-1 (APE/Ref-1), triggering apoptosis based on ROS-dependent disruption of mitochondrial metabolism and activation of p53. Simultaneously, the CRP-Ref1 complex causes transcriptional repression of STAT3, which opposes CRP-induced apoptosis in SMCs [135].

The mitotic activity of SMCs in vivo is small (~ 0.1%) [139], which determines their radioresistance. Another element strengthening the radioresistance is the ability of SMCs to repair DNA damage resulting from the even potentially lethal irradiation doses [140], in which the IR-dependent induction of Ref-1 may play some role [135]. The colonization of some atherosclerotic vessels is associated with the expansion of medial cells making this subset theoretically prone to IR. Such the hypothesis was proved by in vitro studies on cultured SMCs, which exhibited decreased proliferative capacity upon irradiation (1.25–20 Gy) [134, 141, 142] in a mechanism that may involve the induction of cell cycle inhibitors p21 and p53 [143] and the inhibition of growth-promoting STAT3 [135]. These abnormalities may translate to impaired cell reaction to injury, leading to atherosclerotic plaque expansion, restenosis, and vascular hypertrophy [144]. In addition, the irradiated cells displayed reduced contractility [141]. Its weakening was also found in a coculture system when SMCs were grown together with irradiated ECs [65]. In that case, SMCs developed a fibrogenic phenotype manifested by increased mRNA for α-smooth muscle actin (α-SMA), connective tissue growth factor (CTGF), plasminogen activator inhibitor 1 (PAI-1), and collagen III. Notably, developing these traits was not possible in the absence of irradiated ECs. Mechanistically, the formation of the profibrotic phenotype was mediated by the overproduction of TGF-β1 by the irradiated ECs and the subsequent activation of Smad3 signaling in the recipient SMCs [65].

Unfortunately, the literature data regarding radiation’s effects on vascular SMC contractility are conflicting. In contrast to studies showing defective SMCs’ contractility and their profibrotic phenotype, some reports indicate that irradiated animals are characterized by opposite vessel reactions manifested by increased systolic blood pressure [145]. This effect may be related to decreased EC-dependent vessel relaxation (discussed in the previous chapter) and/or increased SMC-dependent vessel contraction. Experiments on animals exposed to whole-body irradiation showed that the vasoconstrictive tendency may be associated with augmented PKC-dependent responsiveness of SMCs’ myofilaments to Ca^2+^. This effect occurs independently of ECs [146]. The PKC also inhibits large conductance Ca^2+^-activated K^+^ channels (BK_Ca_), leading to a depression of an outward K^+^ current that may also, electrophysiologically, contribute to IR-dependent increase in vascular tone and related arterial hypertension [147]. Another group showed that irradiated (10, 20 Gy) SMCs migrate into 3D collagen matrix gels less efficiently, which was accompanied by a reversal of their secretory to contractile phenotype [148]. In the context of these divergent results, one cannot exclude the scenario that the most reliable results derive from animal models, where the SMC-dependent vascular response is associated with the presence of cells having contractile phenotype. In various artificially-created culture systems, this condition is not fully secured.

Experiments performed under controlled in vitro conditions clearly show that the ultimate response of SMCs to radiation is determined by the paracrine, EC-dependent bystander effect [149]. Apart from the earlier discussed adoption of fibrogenic phenotype, the role of neighboring endothelium also appeared to matter for SMCs motility. More precisely, when the SMCs were irradiated alone, the proliferation, as estimated according to the percentage of cells in the S phase of the cell cycle, clearly declined to confirm previously cited findings. Simultaneously, when the non-irradiated SMCs were cocultured with irradiated ECs, their growth capacity unexpectedly increased. Generally, the same happened in the case of SMC migration [65]. SMCs also proliferate more vigorously upon IR treatment in vitro. This observation agrees with examining tissues from patients with cancer undergoing radiotherapy in which SMCs in hypertrophic vessels displayed a higher PCNA index than those in normal tissues [65]. Although the behavior of SMCs undergoing radiation seems to depend strictly on the presence and irradiation of ECs that coexist in blood vessels in vivo, their ultimate reaction is also associated with the IR dose. Namely, when the ECs were subjected to markedly higher radiation (40 Gy), they failed to promote the proliferation of SMCs [150]. Another issue is that SMCs differ in their responsiveness to IR depending on their origin. It was shown in experiments on human aortic and venous SMCs where the latter were less susceptible to radiation (1–50 Gy). However, in both cases, the proliferation upon IR exposure was decreased [151].

### Mechanisms of drug-induced cardiotoxicity and vasotoxicity

Ionizing radiation was the first but not the sole cause of CVS disorders that develop iatrogenically in patients with cancer. Indeed, a much more significant problem and challenge is the cardio- and vasotoxicity induced by the action of various groups of drugs used in oncology, the therapeutic use of which is far broader than irradiation. Cardiac complications in a particular group of high-risk patients are also associated with synergism in the effects of drugs and radiation, as reflected e.g., in the guidelines of the American Society of Clinical Oncology (ASCO) [4].

#### Anthracyclines

**Fig. 2:**
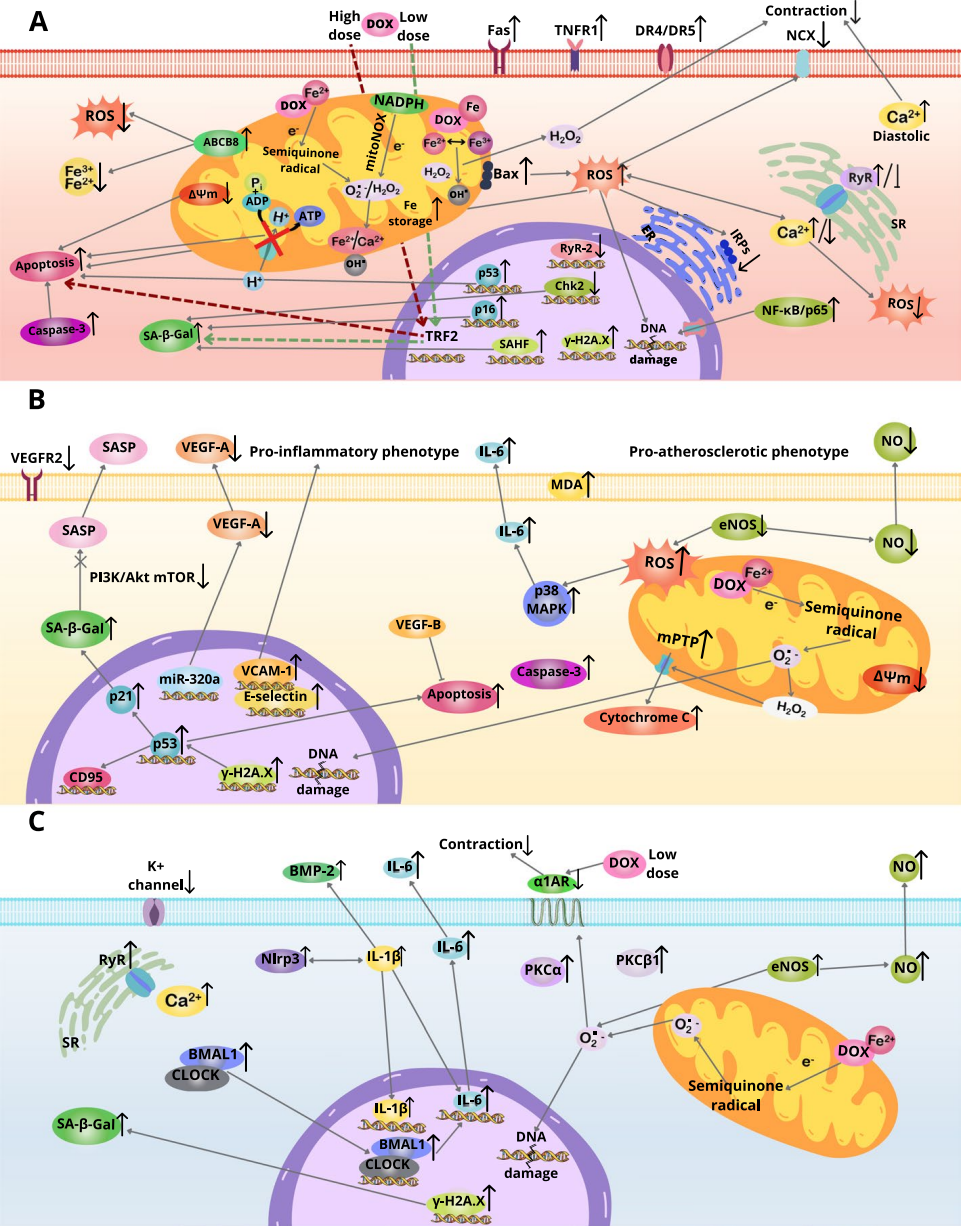
Pathomechanisms of anthracycline-dependent cardio- and vasotoxicity in cardiomyocytes (**A**), vascular endothelial cells (**B**), and vascular smooth muscle cells (**C**). *ER* endoplasmic reticulum, *SR* sarcoplasmic reticulum, *α1AR* α_1A_-adrenoceptor, *CLOCK* *Clock* gene

Clinical observations indicate that anthracyclines predispose oncologic patients to the largest extent to the occurrence of cardiotoxicity that affects their life quality and expectancy, irrespective of oncologic prognosis [152]. Predicting the risk of anthracycline-associated disorders is seriously jeopardized by explicit intraindividual variations in the tolerance of high doses of the drugs, which act dose-dependently [153, 154]. In some individuals, cardiologic adverse effects may occur at a cumulative dose of anthracyclines corresponding to 300 mg/m^2^ or lower. In contrast, other patients have no abnormalities, despite being exposed to doses up to 1000 mg/m^2^ [155]. Population studies showed that anthracycline‐induced cardiotoxicity (ACT) arising as asymptomatic cardiac dysfunction may affect as much as 57% of patients [156]. Restrictive or dilated cardiomyopathy may occur in up to 16% of cases [157]. Patients who received doxorubicin ≥ 250 mg/m^2^ or epirubicin ≥ 600 mg/m^2^, respectively, are defined by the ASCO as those who require intensified cardiomonitoring because of the high risk of developing ACT [4]. Survivors of childhood malignancies subjected to both anthracyclines and radiotherapy are at exceptionally high risk of cardiotoxicity. These patients may experience cardiac events at an early age, and HF may develop in 12.5% of them within 30 years after therapy [158]. In recent years, genetic testing enriching a standard echocardiogram monitoring was proposed to better discriminate between patients with high and low risk of ACT [159]. Proposed cellular pathomechanisms of anthracycline-based cardiotoxicity are depicted in Fig. 2.

#### Cardiomyocytes

Cardiomyopathy is a notorious heart abnormality observed in patients treated with anthracyclines [152]. Classically, the pathomechanism of ACT was linked with increased production and deleterious activity of ROS within cardiac myocytes, considered the main targets of anthracycline toxicity [152]. Furthermore, mitochondrial damage and malfunction are pivotal events underlying ACT. Experiments on several human and animal models revealed that doxorubicin (DOX), which is the most widely used anthracycline [160], is a potent generator of ROS [161–163]. These highly active molecules derive from mitochondria in which the drug incorporates [163], likely due to its strong affinity to phospholipid cardiolipin [164]. 4′-epidoxorubicin (EPI), a synthetic representative of anthracyclines with less pronounced cardiotoxicity [165], is also known to exert its deleterious activity towards cardiomyocytes via elevated ROS. A comparative analysis of mouse HL-1 cardiomyocytes exposed in vitro to DOX and EPI used at 1 μM showed that both anthracyclines stimulate the production of ROS and the accumulation of DNA damage foci (γ-H2A.X). The effects of both drugs are comparable [166]. As a result of ROS accumulation, DOX induces extensive DNA injury, as exemplified in H9C2 cardiomyocytes that accumulate DNA double-strand breaks [167]. Other studies using the same cell type showed that DOX-related DNA injury, namely oxidized pyrimidines and 8-hydroxyguanosine (8-OH-dG), was largely irreparable. This observation contrasts with cell reaction to hydrogen peroxide, where the stressor withdrawal allowed the repair of DNA damage completely [168]. Hearts of DOX-treated mice also possessed an increased level of 8-OH-dG [169]. The insufficient ability of cardiomyocytes to cope with DNA damage after DOX exposure may be associated with the presence of a missense variant rs2229774 (p.S427L) in the retinoic acid receptor gamma (RARG) gene that was found to predispose the cardiomyocytes to reduced effectiveness of DNA damage repair pathways [170]. A recent study on mice and hiPSC-derived cardiomyocytes markedly improved the understanding of anthracycline-related cardiotoxicity by showing that the pathology requires both DNA damage (double-strand breaks) and chromatin damage underlined by histone eviction at specific sites in the genome. At the same time, when the drug variant exerting only chromatin-damaging activity was applied, DOX-related toxic effects were devoid [171].

Mechanisms of increased ROS release upon DOX exposure are complex and proceed in enzymatic and non-enzymatic fashion. Upon its internalization, the quinone moiety of DOX is subjected to one-electron reduction, leading to the generation of the semiquinone radical. When the molecular oxygen is available, the radical transfers to it its extra electron and undergoes reoxidation yielding superoxide anions (O_2_^–^), hydrogen peroxide (H_2_O_2_), and the initial quinone entering subsequent redox cycles. In the presence of transition metals, the primary ROS transform to hydroxyl radical (OH^·^). From the side of mitochondria, NADH dehydrogenase, a component of Complex I of the respiratory chain, was implicated as a final place of DOX reduction and excessive ROS efflux [172, 173]. Another enzymatic factor in DOX-mediated ROS is NADPH oxidase (mitoNOX), which catalyzes one-electron transfer from NADPH to O_2_ [174]. The inhibition of NOX in DOX-treated HL-1 cells results in decreased ROS production, which implies that the one-electron reduction of the quinone ring to the semiquinones originates from the electron donor NADPH utilizing mitoNOX activity [163]. The role of NOX in DOX-related cardiotoxicity was revealed in experiments on mice, in which animals lacking this enzyme were resistant to the chronic adverse effects of this drug [175]. It has also been shown that DOX-related cardiac dysfunction may be orchestrated by topoisomerase IIB (Top2B). Experimental deletion of *Top2b*, the gene encoding Top2, was found to protect cardiomyocytes against DNA damage and altered transcriptome caused by DOX, altogether leading to dysfunctional mitochondria synthesis and ROS release. In animals undergoing this manipulation, the development of DOX-induced progressive heart failure was seriously restricted [176].

Regarding non-enzymatic drivers of DOX-related ROS overproduction, the first potentiating event is an interplay between DOX and iron. The formation of DOX-Fe complex allows the bidirectional transition Fe^2+^-Fe^3+^ in the presence of reducing agents, participating in additional amounts of O_2_^−^ [177]. In the absence of reducing equivalents, the anthracycline-Fe^2+^ complex with H_2_O_2_ yields OH^·^ [178]. Upon DOX exposure, the iron accumulates preferentially in the cardiomyocyte mitochondria. When the mitochondrial ABC protein-B8 (ABCB8) engaged in the iron export [179] was overexpressed in culture conditions and in the hearts of mice, the content of mitochondrial iron declined, along with ROS, and the DOX-related cardiomyopathy was alleviated [180]. Interestingly, there is a vicious cycle in which ROS and iron cooperate, potentiating their effects. Experiments on H9c2 rat embryo cardiomyocytes showed that DOX-dependent ROS participate in cardiotoxicity by deactivating iron regulatory proteins (IRPs) that regulate the fate of mRNAs for transferrin receptors and ferritin [181]. The role of the iron–ROS interplay in DOX-induced cardiotoxicity was confirmed in experiments with the iron-chelating derivative of EDTA, dexrazoxane; the administration of which reduced the iron pool in the cardiomyocytes and restricted its complexing with the drug [182]. Animals that received dexrazoxane before DOX exposure did not develop any clinical, macroscopic, histological, or ultrastructural signs of cardiomyopathy. This was accompanied by decreased ROS and DNA damage and improved mtDNA copy number [183]. Recently, dexrazoxane was shown to mitigate DOX-induced cardiac injury in mice in vivo, which was causatively connected with suppressed apoptosis [184]. In another cardiomyocyte model, dexrazoxane inhibited apoptosis caused by daunorubicin [185]. There is also a report that dexrazoxane inhibited apoptosis in the irradiated (20 Gy) rat cardiomyocytes by down-regulating NF-κB p65 subunit, which corresponded with ameliorated myocardial injury [186]. Mechanistically, the compound appeared to exert this effect by upregulating miR-17-5p, a molecule playing a role in inflammatory reactions and apoptotic cell death [187, 188]. Other research revealed that it prevents DOX from binding to the Top2B complex, which translated to decreased accumulation of DNA double-strand breaks [167]. Till now, dexrazoxane (ICRF-187, Zinecard) is the only cardioprotective drug approved by FDA for preventing ACT [189], because similarly beneficial effects were not observed with respect to other iron-chelating agents [190]. This, in turn, questions to some extent the validity of the iron report hypothesis of DOX cardiotoxicity and suggests that the beneficial effects of dexrazoxane may be related to its impact on molecular prosurviving cellular pathways, rather than to regulation of the iron homeostasis.

Another mechanism of DOX-associated ROS excess involves alterations within Ca^2+^ transporters, including the inhibition of the ions flow via NCX [191], the release of Ca^2+^ via ryanodine receptor (RyR) [161], the activation of L-type channels [192], and the repression of genes coding for Ca^2+^ transporters in SR [193].

The causative role of ROS-dependent pathomechanism of cardiotoxicity positively verified experiments with antioxidative vitamin E, the administration of which reduced DOX toxicity [161]. Similarly, a beneficial effect was observed upon cardiomyocyte preincubation with melatonin, but in this case, this outcome was only temporary and weakened over time [163]. Not all experiments using antioxidants resulted in a reduction of DOX-dependent cardiac injury (e.g., a clinical trial using N-acetylcysteine [194]), indicating that the problem is more unspecific and extends beyond the hypothesized role of ROS as the critical pathogenic triggers responsible for the ACT.

As mentioned earlier, the induction of apoptosis constitutes another pathway contributing to anthracycline-related cardiomyocyte failure [195]. Detailed analysis of this phenomenon indicated that its role in acute cardiac events might be dominant and relatively negligible in the case of delayed cardiomyopathy and HF [196]. Increased incidence of apoptosis, marked by annexin V and the activation of caspases-3/7, was found in HL-1 cells exposed to DOX and EPI [166]. A dose-dependent increase in active/cleaved caspase-3 and Bax was also observed in primary mouse cardiomyocytes treated with DOX [184]. Chromatin condensation and DNA fragmentation to low molecular weight fragments (subG1 fraction), typical signs of apoptosis, were also detected in rodent cardiomyocytes exposed to daunorubicin [185]. In rat cardiomyocytes, DOX-induced ROS are in the mutual interplay with Ca^2+^ liberated from intracellular storages through RyR. When the Ca^2+^ channels in the sarcoplasmic reticulum were blocked, the ROS overproduction in response to DOX was prevented. The same intervention also reduced the extent of apoptosis, as shown according to caspase-3 activation [161]. In H9c2 cells, DOX elicited apoptosis and reduced mitochondrial inner membrane potential (ΔΨ_m_) values, a common feature of apoptotic cells indicating defective course of oxidative phosphorylation and ATP synthesis [197]. Both these phenomena were associated with the activation of p53 [168]. In some cases, however, DOX-induced apoptosis does not require declined ΔΨ_m_, which is the case e.g., in mouse atrial HL-1 cells [163]. Another plausible mechanism of DOX-induced apoptosis was described in human-induced pluripotent stem cell-derived cardiomyocytes (iPS-CMs), in which the drug, as well as other anthracyclines like EPI, daunorubicin, and idarubicin, were found to upregulate a number of death receptors, including TNFR1, Fas, DR4, and DR5, causing apoptosis through binding TNF-related apoptosis inducing ligand (TRAIL) [198].

Apoptosis is not the only kind of cell death affected by anthracyclines. Research on H9c2 cells showed that the drug inhibits autophagy and increases ferroptosis. Both these effects can be prevented by ectonucleotide pyrophosphatase/phosphodiesterase 2 (ENPP2), regulated at a transcriptional level by FOXO4, the level of which was downregulated by DOX [199]. Molecular determinants of DOX-mediated ferroptosis are of particular importance, taking into account the results of other tests that revealed an engagement of this process in DOX-induced cardiomyopathy and mortality in mice and their reversal by ferroptosis inhibition using ferrostatin-1 and iron chelation [200].

Cardiomyocyte autophagy, the altruistic cell death leading to the recycling of cellular components [201], contributes to cardiomyopathy and clinical signs of HF when it is defective [202]. Animals bearing a cardiomyocyte-specific deletion of autophagy-related 5 (Atg5), a protein engaged in the extension of the phagophore membrane in autophagic vesicles, suffer from dilated cardiomyopathy in adulthood [202]. In H9c2 cardiomyocytes, DOX inhibits autophagy by activating E2F1/mTOR complex 1 (mTORC1) signaling, which is followed by apoptosis triggered by E2F1/AMP-activated protein kinase α2 (AMPKα2) pathway [203]. However, the observations regarding autophagy are ambiguous. For example, DOX stimulated autophagy in neonatal rat ventricular cardiomyocytes and this action was linked with a depletion of GATA4 transcription factor, the positive regulator of autophagy-related genes [204]. One may speculate that the disparities in the perception of DOX impact on autophagy may be interpretive and related to the complexity and multi-stage mechanism of autophagy. Some compromise may come from studies showing that DOX impairs autophagic flux in cultured cardiomyocytes in vitro and mice in vivo, simultaneously promoting the accumulation of noncompetent autolysosomes. The autophagic flux is limited because DOX inhibits lysosome function by alkalization of their pH via suppressing vacuolar H^+^-ATPase, which eventually prevents autolysosome cargo processing. When the very first events in the autophagic cascade were attenuated, DOX-related cardiotoxicity was diminished. And conversely, when the initiation of autophagy was intensified, cardiotoxic drug effects were exacerbated. As such, the direction of events may indicate that autophagy may protect against DOX-mediated cardiac injury, only when the autophagic flux is efficient. When dysfunctional autolysosomes accumulate and the process cannot proceed further, pathological cardiac remodeling based on ROS activity occurs [205].

Experiments on H9c2 cells followed by studies on primary neonatal cardiomyocytes showed that DOX induces their senescence, marked by the presence of SA-β-Gal, senescence-associated heterochromatin foci (SAHF), and elevated p16 [206]. Neonatal rat cardiomyocytes exposed to DOX displayed increased formation of ROS and SA-β-Gal activity, with no concomitant increase in apoptosis, despite activated p53. The development of the senescence phenotype likely took place instead of entering apoptosis, because the concentration of DOX was relatively low and there was no urgent need to eliminate the affected cells [207]. This assumption agrees with another report in which cardiomyocytes exposed to 0.1 μM of DOX exhibited signs of senescence, whereas their counterparts treated with 1.0 μM of DOX became apoptotic [208]. Similar trends were found in cardiomyocytes exposed to low and high doses of EPI [209]. Intriguingly, cells that approached senescence evolved towards hyperploidy, a downregulation of DNA damage-induced checkpoint kinase Chek2, and chromosomal disturbances, which may indicate the initiation of mitotic catastrophe [210]. Cardiomyocytes subjected to lower and higher doses of DOX displayed diversified changes in telomere binding factors TRF1 and TRF2 at both mRNA and protein levels, and changes in TRF2, regulated by p38 MAPK, were critical for the acquisition of senescence or apoptotic phenotype. The low magnitude of TRF2 downregulation caused senescence in the p53-dependent mechanism and the high magnitude of TRF2 down-regulation caused apoptosis in the p53-independent fashion [208]. DOX-induced senescence of neonatal rat ventricular myocytes and H9c2 is also regulated at the transcriptional level by PPARδ sequestering the transcriptional repressor protein B-cell lymphoma-6 (Bcl6). When PPARδ was experimentally inhibited, the level of Bcl6 increased via p38 MAPK, JNK, and Akt activation, followed by amelioration of the senescence phenotype [211].

One of the elements of DOX-induced cardiomyocyte senescence is an abnormal pattern of troponin phosphorylation that may lead to inefficient cardiac contraction [207]. In fact, another aspect of cardiomyocyte functioning that may be affected by anthracyclines is their contractility [212]. Either DOX or EPI (10 μM/20 min) depress several indices of contractility, including the maximum extent of cell shortening, peak shortening velocity, and peak relaxation velocity of single twitches. The magnitude of effects related to both drugs was comparable. The adverse effects were reversible by cell protection by catalase (CAT), and only partly by superoxide dismutase (SOD), which was suggestive of the role of H_2_O_2_ as the central culprit of this abnormality [213]. DOX-related disturbances in cell contractility are strongly connected with the distribution and the transient of Ca^2+^ using transporters associated with systolic Ca^2+^ signal. Research on rodent cardiomyocytes showed that RyR, responsible for Ca^2+^ leak from SR [214], is an early target of DOX and its activation underlies an increase in diastolic Ca^2+^ and the decrease of caffeine-induced Ca^2+^ peak amplitude [163]. At the same time, decreased caffeine-induced Ca^2+^ transient decay rate upon DOX exposure was connected with an inhibition of the NCX activity, which may explain diastolic Ca^2+^ overload and the successive contractility malfunction [163].

The last, but not the least, element of ACT development is local inflammation [215]. Histopathological examination of hearts isolated from mice treated with DOX showed that a large amount of inflammatory cells were accumulated in the disordered tissue [184]. DOX-treated adult mice also displayed cardiac fibrosis that was accompanied by the accumulation of macrophages M1 and increased levels of NF-κB p65 subunit, with a trend toward increased production of the tumor necrosis factor receptor 2 (TNFR2). At the same time, IL-6 level was decreased, and the levels of tumor necrosis factor (TNF-α) and IL-1β were unchanged [216]. The chemotaxis and the accumulation of the immune cells are mediated by several cytokines overproduced in response to DOX in a mechanism involving activation of p38 MAPK and NF-κB [217, 218]. The role of IL-6, a potent proinflammatory cytokine, in the ACT is elusive. Decreased cardiac production of IL-6 upon DOX exposure [216] may imply that it may exert a cardioprotective function, which is in line with the observation that the knockdown of IL-6 in cultured cardiomyocytes led to the augmentation of DOX-induced apoptosis. This picture is, however, blurred by the observation that plasma levels of IL-6 mRNA positively correlated with the severity of ACT [219]. The unclear and hard-to-interpret direction of changes in DOX-related production of proinflammatory factors may depend partly on the research model used, including in vitro and in vivo tests. This is demonstrated e.g., in the case of H9c2 cardiomyocytes, whose treatment with DOX led to increased production of IL-1, IL-6, and TNFα in NF-κB/p65-dependent manner [220].

#### Vascular endothelial cells

Endotheliotoxicity of anthracyclines is the next piece of the puzzle constituting ACT and the coronary endothelium, and peripheral blood vessels are the next structures affected by these drugs [221]. Among several traits of vasotoxicity, the DOX-related induction of oxidative stress seems to be the most evident one. Mice subjected to DOX exhibited thoracic aorta damage, as evidenced according to inflammatory infiltration, cell swelling, and interstitial cell hypertrophy. This picture coincided with impaired vascular responsiveness, as shown by quantifying endothelium-dependent dilation [222], decreased density of cardiac capillaries, and increased permeability of cardiac microvessels [223]. At a cellular level, DOX reduced the viability of HUVECs, likely due to the induction of p53-dependent apoptosis (subG_1_ cells) [224]. Mechanistically, the apoptosis was triggered by activated p53, which propagated the signal to its downstream target CD95 in a transcriptional mechanism.

Moreover, DOX activated executioner caspases, as shown by the proteolytic cleavage of the PARP nuclear substrate and the activation of caspase-3, albeit the caspase activation was disconnected from CD95 signaling. Intriguingly, DOX-related apoptosis was determined by culture density: it was present in low-density cultures and absent in the confluent ones. This observation may explain the antiangiogenic activity of DOX, which appeared to trigger the elimination only of proliferating cells that, in contrast to their resting counterparts, actively contribute to tumor neovascularization [224]. Another group also reported markedly higher sensitivity to DOX of dividing than resting ECs, concerning cell proliferation [225].

Several abnormalities caused by DOX were found within mitochondria, whose function associated with respiration and glycolytic activity was impaired. The production of mitochondrial ROS, along with the cellular species, was intensified, which coincided with reduced ΔΨ_m_ values, depressed expression of eNOS, and decreased NO content. Mitochondria in DOX-treated HUVECs play a dual role as the source and the victim of ROS. They are clearly disrupted by ROS, which is manifested by their swelling, opening permeability transition pores (mPTP), and efflux of cytochrome C to the cytosol. At the same time, the released ROS may be absorbed by nearby intact mitochondria, leading to damage propagation in a positive feedback reaction, ultimately initiating an apoptotic cascade. Significantly, negative changes elicited by DOX were reversed by edaravone, a free radical scavenger, supported by an mPTP closing agent. The endothelial function, including cell ability to generate NO was restored, in turn, by edaravone and by the adenovirus pAD/eNOS delivery, clearly reinforcing the causal role of oxidative stress and NO deficit in DOX-related EC dysfunction [222]. Other authors showed that DOX undergoes a reductive activation at the reductase domain of eNOS, generating the semiquinone and O_2_^−^, at the expense of reduced production of NO. In this context, the DOX-related cardiotoxicity was attributed to a dynamic dose-dependent switch in the enzymatic activity of eNOS, which may change from a NO-generating enzyme (eNOS activity) to a ROS-generating enzyme (NADPH oxidase activity) [226]. These observations may clarify potentially confusing findings e.g., those derived from experiments on bovine aortic endothelial cells (BAEC). In these cells, DOX increased eNOS transcription and protein activity, and cell preincubation with antisense eNOS mRNA resulted in decreased incidence of DOX-induced apoptosis [227].

The synthesis of NO by ECs is regulated, at least to some extent, by specific miRNA. DOX was found to increase the expression of miR-320a in the hearts of drug-treated mice and in HUVECs in vitro. The attenuation of this molecule translated to improved heart vessel density in vivo, repressed endothelium damage, and inhibited cardiac failure. In culture conditions, miR-320a silencing improved cell proliferative capacity, reduced the frequency of apoptosis, and relieved DOX-induced decline in NO synthesis [189]. Target prediction analysis revealed that vascular endothelial growth factor A (VEGF-A) is one of the theoretical miR-320a targets. Further studies confirmed the negative relationship between these two molecules, which may explain decreased VEGF-A protein levels in hearts after DOX exposure. A reduced level of VEGF-A was also found in vitro in DOX-treated rodent ECs [228]. VEGF-A contributes to DOX-associated deterioration of EC function, as its knockdown resulted in weakened cell proliferation, migration, tube formation, and decreased NO release [189]. The potent role of the second variant of VEGF, that is VEGF-B, in DOX-related cardiotoxicity revealed experiments on mice whose DOX-induced cardiac dysfunction was entirely alleviated by delivery of adeno-associated viral vector expressing VEGF-B. Moreover, in cultured ECs, VEGF-B inhibited DOX-related apoptosis and restored their ability to form capillaries [229].

DOX-related abnormalities in VEGF activity are connected with the development of ACT, and EC senescence seems to play a role in this process. HUVECs exposed to DOX display reduced VEGF receptor type 2 (VEGFR2) production, acting as a tyrosine kinase receptor in ECs, in keeping with observations on DOX-treated rat cardiac microvascular ECs [228]. This alteration was accompanied by the acquisition of senescence phenotype manifested by the activation of SA-β-Gal, p53, and its downstream effector, p21, and deterioration of growth-promoting proteins, cyclin A and lamin B1. At the same time, conversely to the report cited above [189], which was made on the same cell type but using a different dosage regimen (250 nM/24 h vs. 5 μM/12 h), DOX transiently induced VEGF-A at mRNA level. When the cells became senescent, the VEGF-A mRNA expression returned to values typical for untreated cells. The VEGFR2 protein dynamically increased, but did not reach the steady-state values before DOX exposure. The development of senescence phenotype in DOX-treated HUVECs also coincided with increased autophagic flux. Autophagy-related VEGFR2 trafficking and degradation did not turn out to be the reason for the protein decrease upon DOX. The actual cause was the inhibition of the protein synthesis by impinging on the p53-mTOR signaling [230]. At the same time, ECs that senesced upon DOX exposure do not display typical SASP-related overproduction of proinflammatory cytokines, which appeared to be a self-limiting phenomenon in vitro and in vivo. Namely, DOX stimulated transient hypersecretion of IL-6 in a mechanism engaging ROS-dependent activation of p38 MAPK, but it was recognized as an acute secretory profile, distinct from canonical SASP. When the drug induced HUVEC senescence, the SASP could not develop due to the suppression of PI3K/Akt/mTOR axis [231]. Recent studies in which DOX-induced senescence of human aortic ECs was prevented by vitamin D3 administration also pointed to the role of IL-10 being controlled by pAMPKα/SIRT1/FOXO3a signaling complex [232]. Apart from upregulated IL-6, the proinflammatory phenotype of DOX-treated ECs also includes increased expression of VCAM-1 and E-selectin, contributing to increased adhesion of neutrophils to these cells [225].

An integral element of cellular senescence and DOX-related toxicity is DNA damage, the spectrum of which is broad and includes DNA strand breaks, induction of DDR (γ-H2AX), and oxidative modifications [233, 234]. A comparative analysis of DOX and daunorubicin concerning their ability to destroy EC DNA showed that both these analogs are genotoxic, albeit the effects caused by the latter are more pronounced [235]. DOX exerts its deleterious activity also towards cellular lipids, as evidenced according to increased concentration of malondialdehyde (MDA) in treated HUVECs [234].

#### Vascular smooth muscle cells

SMCs are the second cellular component of blood vessels violated by anthracyclines and thus participating in the ACT. Upon exposure to DOX, they prematurely senesced, displaying hypertrophic appearance, specific markers, and activated DDR [236]. In rat aorta SMCs, the drug deregulates oxidative stress-related parameters [increased ROS production and decreased catalase (CAT)] and intensifies eNOS-NO axis, which coincides with up-regulated PKCα and PKCβ1 [237]. DOX-induced senescence may also involve, apart from exacerbated oxidative stress, increased production of urokinase receptor (uPAR), whose experimental silencing in either in vitro or in vivo conditions abrogated the drug-induced senescence. The prosenescence activity of uPAR was linked with ubiquitination and proteasomal degradation of TRF2 [238].

Research on isolated arteries showed that DOX used at a relatively low dose (0.3 μM) affects SMC functioning by decreasing noradrenaline-induced contractions, which was associated with a depressed induction of α_1A_-adrenoceptor protein, but not of mRNA. The tissue exposure to SOD partially prevented these abnormalities, suggesting that O_2_^−^ produced during redox cycling of DOX downregulates the receptor at the protein synthesis level. When the dose of DOX increased to 1 μM, the signs of apoptosis approached, whereas at 10 μM the cultured arteries became necrotic [239]. In addition, experiments on DOX-treated mice or those using mouse aortic segments exposed to this drug (1 μM) ex vivo revealed decreased phenylephrine (PE)-induced SMC contraction, due to a depressed tonic phase of contraction characterized by Ca^2+^ influx. At the same time, DOX did not change the transient PE contraction related to Ca^2+^ release from the SR [240]. A systemic administration of DOX into BALB/cJ mice led to detrusor smooth muscle hypertrophy, increased voiding frequency, and a substantial attenuation of the muscle contractility, followed by a slower relaxation. Unlike the cardiovascular effects of DOX, in the case of urinary system dysfunction, the results of the drug were not associated with oxidative stress but with a reduced amount of large-conductance Ca^2+^-activated K^+^ channels and suppressed myosin light-chain phosphorylation [241].

DOX also affects lymphatic muscle cells. It leads to a dose-dependent inhibition of rhythmic contractions, remarkably reducing the flow. This effect was paralleled by a tonic rise in cytosolic Ca^2+^ level, which occurred via the opening of RyR and resulting calcium leak from SR. This discovery reveals that pharmacological blocking of RyR-dependent Ca^2+^ inflow may be a helpful strategy in reducing DOX-related lymph vessel injury and the development of lymphedema [242].

A substantially different view on contractility and Ca^2+^ management in SMCs treated with DOX delivered research on cells from C57BL/6 mice, in which the drug intensified both phasic and tonic contractions in denuded vessels and elevated levels of [Ca^2+^]_i_ in single muscle cells. The study showed, however, how vital the dose of DOX concerning contractile SMC action is. When used at 100 μM, it increased rhythmic contraction amplitude, but when the dose was 1 mM, the amplitude was abolished with an increase in maximal tension [243]. A diminished relaxation of aorta SMCs in response to NO donor, sodium nitrate, was also found in rats treated with DOX. This effect coincided with an induction of cellular senescence, manifested by increased SA-β-Gal, p16, p27, IL-6, and IL-8 [244].

The current view on the proinflammatory phenotype of DOX-treated SMCs attributes this phenomenon to cellular senescence. Primary rat aortic SMCs subjected to DOX become senescent (SA-β-Gal, γ-H2A.X, p21) [245]. This coincided with stimulation of Nlrp3 inflammasome, leading to an upregulated secretion of IL-1β but not, surprisingly, IL-6. The increased IL-1β has been recognized as a mediator of calcification, which appeared to be preventable in vivo in DOX-treated Nlrp3^−/−^ mice. Further research showed a proinflammatory autostimulatory loop in which IL-1β promotes IL-6 mRNA and mRNA for itself, as well as induces the Nlrp3 and the osteogenic marker BMP-2 [245]. Increased mineralization may be another pathogenetic element contributing to increased vessel stiffness and related proatherosclerotic vessel remodeling. The matter of DOX-related vessel stiffening resulting from changes in SMC behavior is, however, still elusive as a recent study showed that it occurs ex vivo translating to decreased cell contractility. In contrast, in vivo this effect was absent [240].

Increased inflammatory response of SMCs e.g., an elevated IL-6 mRNA may also be connected with the activity of brain and muscle Arnt-Like protein-1 (BMAL1), a molecular promotor of SMC proliferation [246], was simultaneously upregulated by DOX. Paradoxically, the overexpressed IL-6 was further raised upon BMAL1 silencing, which also impaired cell viability and increased activity of antiinflammatory nuclear factor erythroid 2-related factor 2 (Nrf2), increased NADPH oxidase 4 mRNA, and increased phosphorylation of p38 MAPK. The causative engagement of activated p38 MAPK in DOX-related inflammation was confirmed by intervention tests in which chemical inhibition of the kinase translated to a deterioration of BMAL1-dependent elevation of IL-6 [247].

### Platins

Although the primary adverse events of cancer therapy when using high-dose of platin-based drugs, including cisplatin (CIS) and carboplatin (CPT), include nephrotoxicity, neurotoxicity, ototoxicity, and gastrotoxicity [248, 249], there are also reports pointing to the harmful activity of platins in disturbances encompassing CVS [250, 251]. Much broader evidence concerning potential cardio- and vasotoxicity stem from basic research on cellular models uncoupled from clinical observations.

#### Cardiomyocytes

Arrhythmias and other CVS disorder manifestations experienced by cancer survivors treated with platins [252–254] are causatively connected with the malfunction of cardiac cells. The hearts of animals subjected to CIS are hypertrophic and bear disarrayed cardiac muscle fibers architecture, which corresponds with cardiac dysfunction. The reversal of this trait by exogenous flavonoid kaempferol, known for its antiinflammatory and antioxidative properties [255, 256], suggests the plausible pathomechanism of platin-induced toxicity [257]. Rats exposed to CIS exhibited decreased diastolic blood pressure with unaltered systolic blood pressure and heart rate. At the highest dose of CIS (3 mg/kg), the drug decreased left ventricular developed pressure (LVDP) and increased end-diastolic pressure (EDP) values [258]. An aberrant cardiac histology concomitant with elevated apoptosis is also a consequence of CPT administration [259].

In culture conditions, rat cardiomyocytes exposed to this drug became apoptotic, as shown by increased caspase-3, 9, and cytochrome c, and decreased Bcl-xl. The inhibition of apoptosis incidence in cells pretreated with ROS scavenger, N-acetylcysteine, demonstrated the association between CPT-induced apoptosis and oxidative stress [259]. H9c2 cells exposed to CIS display increased apoptosis, as evidenced according to the increased percentage of TUNEL-positive cells, upregulated expression of Bax, and downregulated expression of Bcl-2. At the stage of apoptosis effectory pathway(s), STING signaling may play a role. CIS enhanced this pathway, and its silencing prevented drug-induced cell death [257]. The translocation of proapoptotic Bax from the cytoplasm to the mitochondria was essential in CIS-induced apoptosis [260]. Other apoptosis indices found in rat ventricular cardiomyocytes subjected to CIS include ΔΨ_m_ depolarization and increased leak of cytochrome c from mitochondria to cytosol, confirming the mitochondrial mechanism of apoptosis [261]. An induction of transcription factor Nrf2 and heme oxygenase 1 (HO-1) mitigating CIS-related apoptosis in H9c2 cells delivers other targets involved in the cardiotoxicity of platins and points to the causative role of oxidative stress [262].

Indeed, decreased expression of Nrf2, which protects cells against oxidative stress [263] and reduced activity of HO-1, an enzyme contributing to Nrf2 pathway activation [264], was described in CIS-treated cardiomyocytes. Cardiomyocytes exposed to this drug display signs of oxidative stress, including increased production of ROS, depleted SOD, CAT, and reduced glutathione (GSH), and increased storage of MDA. In addition, CIS upregulates p38 MAPK and ERK1/2 [260, 265]. Experiments with growing doses of CIS showed that the drug stimulates the phosphorylation of ERK1/2 dose-dependently and that the kinase inhibition translates to decreased cell propensity to apoptosis [260]. As per p38 MAPK, the p38α makes cardiomyocytes more vulnerable to apoptosis evoked by various triggers via upregulation of the proapoptotic Bax and Fas and downregulation of ERKs and Akt prosurvival pathways [266, 267].

Immunohistochemical evaluation of myocardial tissues from CIS-treated mice showed the formation of a proinflammatory environment, exemplified by the tissue infiltration with macrophages. This observation was linked with increased mRNA and protein for TNFα and IL-6, and mRNA for MCP-1, likely contributing to monocyte chemotaxis. NF-κB induced transcription of the above genes, and CIS stimulated its activating phosphorylation [257].

Mitochondrial malfunction and cardiomyocyte apoptosis may also underlie disturbances in cardiac muscle contraction. Experiments on C57 mice showed that CIS (10 mg/kg per day) led to the development of myocardial contractile dysfunction, as evidenced by decreased LVDP. This alteration coincided with ultrastructural abnormalities within the mitochondria, the activation of the ER stress response, and apoptosis in CIS-treated cardiomyocytes [268].

#### Vascular endothelial cells

Platin drug-related damage to ECs predisposes cells to various pathogenetic events, including thrombotic disease, atherosclerosis, and disturbed vascular contractility. In young cancer survivors, delayed complications of CVS may occur 10–20 years from the point at which the therapy was terminated [269]. When it comes to acute cardiotoxicity of platins, it is generally linked with direct damage to ECs, as hypothesized according to the observation of increased release of von Willebrand factor antigen (vWF:AG) [270], the marker of EC injury [271]. Increased incidence of thromboembolic events may also be associated with upregulated production of procoagulant endothelial microparticles (EMPs) by HUVECs and human pulmonary microvascular ECs. The EMPs promoted thrombin generation in a tissue factor-independent mechanism, which coincided with augmented cell death [272]. Direct toxicity of platins may also lead to the deregulation of vascular tone. Experiments focused on the contractile action of thoracic aortic rings obtained from Sprague–Dawley rats revealed that CIS counteracted KCl- and phenylephrine-stimulated contractions in both endothelium-intact aortic rings and endothelium-denuded aortas. Subsequent tests on cultured HUVECs showed that these abnormalities could be associated with CIS-related inhibition of ATP-induced intracellular Ca^2+^ concentration burst [273].

CIS toxicity also translates to structural abnormalities within the endothelium in vivo, including vacuolation, subendothelial swelling, and internal elastic membrane decomposition. All these negative changes were prevented in animals treated with vitamin E, which implies the contribution of CIS-related oxidative stress [274]. Research on cultured cells (HUVECs) showed that CIS both intensifies the production of ROS and decreases GSH/GSSG ratio compared with untreated cells [275].

Another critical sign of platins’ endotheliotoxicity, likely associated with ROS activity, is DNA damage. When it comes to cancer cells, CIS and CPT bind to the N7 reactive center on purine bases, causing DNA injury that stops replication and turns cancer cells towards apoptosis and/or necrosis [276, 277]. In the context of ECs, CIS inhibited transcription reaching the highest inhibitory effect 24 h after exposure. The products of the drug activity included DNA intrastrand cross-links (i.e., 1,2-GG, 1,3-) and interstrand cross-links, which derive from monoadducts with a slow kinetic, which are the most potent transcription blockers. In addition, CIS caused a massive accumulation of γ-H2AX and 53BP1 foci found in a significant subset of cells 24 h after the exposure, followed by minor phosphorylation of ATM, Chk1, and p53 [278].

Because apoptosis is often a consequence of ROS activity and DNA damage, this aspect of EC biology was tested in cells treated with platins. Experiments on HUVECs showed that CIS induces apoptosis (TUNEL and cleaved caspase-3) dose-dependently, accompanied by decreased Bcl-2 level. Moreover, CIS upregulates hypoxia-inducible factor-1α (HIF-1α), which positively regulates angiogenesis-related genes at the transcriptional level [279], although the production of VEGF remained unchanged. Instead, CIS intensified the interaction of HIF-1α with p53 indicating that this transcription factor may act as a switch directing cells toward p53-dependent apoptosis rather than as a regulator of EC survival and angiogenesis [280]. CIS-treated apoptotic ECs were characterized in vitro by a morphological depopulation, as evidenced according to decreased transendothelial electrical resistance and repressed expression of tight junction proteins, occludin and zonula occludens-2 (ZO-2) [281].

One of the aspects of EC biology most affected by platins is their motility, being a somewhat simplistic measure of angiogenesis [282]. It must be stressed, however, that literature data regarding the modulation of angiogenesis by platins concern tumor vasculature to the most considerable extent; thus, they are basically uncoupled with potential cardio- and vasotoxicity. CIS inhibits dose-dependent proliferation and tube formation in HUVECs in vitro, as well as rabbit corneal neovascularization in vivo [280, 283]. Furthermore, the drug inhibits HUVEC migration and upregulates the activation of p38 MAPK and JNK signaling routes. Intervention tests directed towards the understanding of the mechanism of the attenuated migration showed that it was associated with decreased production of extracellular matrix (ECM) degrading enzyme, matrix metalloproteinase 2 (MMP-2) [284]. Another research project extended these findings by showing that CIS inhibits vascular endothelial growth factor (VEGF)-mediated angiogenesis in Matrigel™ plugs in mice [285]. Interestingly, the inhibitory effects of CIS on HUVEC motility may proceed not only after the direct exposure, but also indirectly in a paracrine manner. This assumption confirms research on HUVECs, whose migration and tube formation capacity was unaltered upon CIS applied at doses between 0.01 and 1 µM. Conversely, when the HUVECs were exposed to a conditioned medium generated by CIS-treated lung cancer cells, the angiogenic behavior of ECs remarkably declined. Mechanistically, this effect was coupled with the activity of cancer cell-derived tissue inhibitor of matrix metalloproteinases-1 (TIMP-1) interacting with p38 MAPK and ERK1/2 signaling pathways [286].

In the case of CPT, its angiogenesis-modulating action seems more complex. Namely, the drug potentiates the production of angiogenesis-promoting VEGF by ECs, and the neutralization of the secreted VEGF led to their sensitization to the drug and massive apoptosis. Interpreting these results, authors of the study postulate that endothelium-derived VEGF is a survival agent that CPT induces to protect tumor vasculature during therapy [287].

#### Vascular smooth muscle cells

The existing literature provides ambiguous data regarding platin-related changes in blood vessel contractility, which may be associated with different experimental models used (e.g., different species of rats) and different dosage regimens in vivo and in vitro. Experiments on rats showed that their chronic exposure to CIS did not alter the aortic vasoconstrictive reaction to phenylephrine, but suppressed the carbachol-related vasorelaxation [258]. On the other hand, the research cited above was in the context of EC dysfunction and was performed using the same model indicated that CIS inhibits contractions in endothelium-denuded aortas [273], which may suggest the engagement of other cell populations, likely SMCs. However, direct exposure of SMCs to platins has never been tested; thus, their role in vascular reactions still has to be experimentally established. At the same time, there is a risk that functional changes within SMCs may be hard to interpret as they display, as shown using the rat model, high heterogeneity in some biological reactions, specifically proliferation and apoptosis, to external stressors, including CIS [288]. However, some clues regarding plausibly adverse effects of such exposure may be derived from research on rabbits. Intraarterial administration of high-dose of CIS resulted in damage to the tunica media, including vacuolation and disorganization of SMCs, fibrinoid necrosis, and hemorrhage [289].

### Taxanes

In cancer therapy, taxanes, especially paclitaxel (PCT) and docetaxel (DCT), hyperstabilize cytoskeletal microtubules that jeopardize various aspects of cancer cell proliferation, particularly chromosome segregation during mitosis [290, 291]. Their effectiveness against several malignancies goes hand in hand with side effects on healthy organs, including the CVS [292]. At the same time, unlike other drug groups with conclusively cardiotoxic activity, the outcomes of taxane administration appear to be more complex and both negative and positive. As per cell types discussed in this article, adverse consequences of taxanes are most noticeable in the case of cardiomyocytes and ECs. Regarding vascular SMCs, PCT activity is used to prevent some CVS disorders e.g., restenosis occurring after percutaneous coronary interventions (PCIs) in patients with coronary heart disease. In addition, cardio- and vasotoxicity of taxanes extended pathophysiologically beyond the cellular context and engaged some indirect effects associated with the excessive histamine release [293, 294]. In response to histamine receptor stimulation, cells, including cardiomyocytes, require more oxygen, which leads to cardiac vessel contraction, contributing to the development of their arrhythmogenic phenotype [295].

#### Cardiomyocytes

The development of arrhythmias is often associated with taxane therapy, and an abnormal Ca^2+^ metabolism is the probable cause of this disturbance [292, 296]. Experiments on the guinea pig model revealed that spontaneous Ca^2+^ oscillations might lead to premature heartbeats, ventricular tachycardia, or ventricular fibrillation [297]. Neonatal rat ventricular cardiomyocytes subjected to PCT (at 800 ng/ml, equivalent to concentrations used in the treatment of patients with cancer) are characterized by an increased frequency of spontaneous Ca^2+^oscillations [298]. Importantly, this effect was uncoupled from either the primary mode of PCT action affecting microtubules or its secondary result—apoptosis. The prooscillatory impact of PCT was sensitive to prevention by inhibiting the inositol-1,4,5- trisphosphate receptor (InsP_3_R), which promotes Ca^2+^ release and interacts with neuronal calcium sensor 1 (NCS-1), and is also upregulated by the drug in vitro and in vivo. At the same time, it was independent of RyR, thus playing an essential role in Ca^2+^ efflux from SR [299], which shows, in turn, that PCT does not influence SR Ca^2+^ reservoir [298].

Cardiac ischemic events are the next type of taxane-related CVS disorders [300], and hypoxia plays a vital role in their pathophysiology. Deficits in oxygen delivery cause disturbances in cellular energy metabolism that translate to several structural and functional anomalies [301]. This state rapidly stimulates glycolysis in cardiomyocytes, providing anaerobic ATP and lactate in a process controlled by HIF-1α [302]. In addition, cellular microtubules, playing a pivotal role in the transmission of mechanical forces within the myocardium, are disorganized in hypoxic conditions [303]. Their breakdown level determines cardiomyocyte viability [304]. The outcomes of PCT action on cardiomyocytes under hypoxia depend on its concentration, experimental model, and analyzed cardiac function. High doses of this drug disrupt the microtubular structure, which is accompanied by decreased HIF-1α nuclear content, reduced cell viability (elevated LDH—a measure of a cell injury), downregulated glycolytic enzymes (phosphofructokinase, pyruvate kinase), and depleted ATP. However, when PCT was applied at low doses, the direction of all these effects was the opposite. The drug counteracted hypoxia-related microtubule breakdown and ameliorated energy metabolism and cell viability by increasing HIF-1α content [304]. The above findings suggest that under specific conditions (read: at relatively low doses), PCT may prevent microtubule disruption and, thus, potentially reduce cardiac infarct size. Analogically, DCT decreased LDH release and oxidative stress in confluent cultures of newborn rat cardiomyocytes submitted to cold ischemia–reperfusion [305]. Furthermore, PCT used at doses of 0.3–3 μM enhanced heart rate, LVDP, and work index in isolated rat and rabbit hearts subjected to local ischemia without reperfusion [306]. Experiments on isolated rat hearts showed that PCT used at relatively low doses of 0.1–1.0 μM helped to preserve microtubule structure in ischemic conditions. This effect was coupled with decreased ROS production and increased mitochondrial electron transport chain complexes I and III activities. In addition, PCT increased the phosphorylation of JNK1, which appeared to mediate an elevation in HO-1 expression [307]. Another plausible mechanism of PCT-dependent protection of cardiomyocytes against ischemic ventricular arrhythmias includes restoration of gap junctional protein, connexin 43 (Cx43) [308].

At the same time, other research on guinea pig hearts showed detrimental, arrhythmogenic effects of PCT [309]. In isolated rat hearts maintained under normoxic conditions, PCT exerted a cardiodepressive activity, manifested by decreased dose-dependent heart rate and LVDP, and increased perfusion pressure. At the same time, it failed to reduce infarct size during ischemia, despite its protection against hypercontracture. This lack of protective effect coincided with a higher increase in cytosolic Ca^2+^ content during ischemia, which may explain the absence of the drug reactivity against infarction observed in isolated hearts. These findings may imply that PCT intensified injury during ischemia, initiating rupture of the sarcolemma in isolated cells, with secondary loss of intracellular cargo, including Ca^2+^, and the consequent suppression of cell hypercontracture during reperfusion [303]. Last, but not least, the profound increase in perfusion pressure triggered by PCT, along with highly contextual changes remaining, may translate to diversified clinical consequences in oncologic patients. They may be expressed as brady- and tachyarrhythmias, atrioventricular conduction block, or myocardial ischemia and infarction [292, 310].

As shown before, PCT exerts antioxidative activity by modulation of mitochondrial metabolism in rat [307] and rabbit ischemic hearts [311]. The impact of taxanes on oxidative stress-related parameters is more complex and probably associated with an oxygenation tissue status. Under normoxia, PCT induced oxidative stress in adult Wistar rats, as evidenced according to increased concentration of lipid peroxidation product, MDA, and NO. Moreover, cardiac damage was found, including diffused edema, hemorrhage, congestion, hyaline exudates, and necrosis [312]. In H9c2 cells, DCT stimulated ROS production, followed by a compensatory increment of mRNA expression for CAT and GPX, and protein for SOD1. Intensified endoplasmic reticulum stress (increased ATF6 and CHOP) and decreased cell viability, due to increased incidence of apoptosis and necrosis, accompanied this imbalance in redox status [313].

#### Vascular endothelial cells

Taxanes exert antiangiogenic activity, primarily when used at low concentrations [314]. Experiments on C57BL/6N mice showed that PCT suppresses angiogenic reactions stimulated by cancer-derived conditioned medium inserted in a pellet of Matrigel™ injected subcutaneously (s.c.). Consequently, the drug dose-dependently inhibited various aspects of angiogenesis in vitro, including HUVEC proliferation, migration, invasiveness, and capillary network formation [315, 316]. This antiangiogenic activity of PCT seems to be a universal phenomenon, as it was present in various cellular models, such as HUVECs [315, 316], HDMECs [317], and human microvascular ECs (HMEC-1) [318]. Research conducted on HUVECs and HMEC-1 cells revealed that PCT inhibits angiogenesis by intensifying microtubule dynamicity, manifested by increased growth, shortening rates, and extents. This impact coincided with decreased anaphase/metaphase ratio and was uncoupled from apoptosis [318]. Further research on HUVECs and HMVEC-1 cells showed that PCT inhibits their proliferation in two distinct mechanisms, determined by its dose. Namely, the drug exerts primarily cytostatic activity at low concentrations, whereas at higher doses (> 10 nM), it becomes cytotoxic. The inhibition of EC proliferation occurs exclusively at cytostatic doses, does not involve any structural microtubule modifications, and is based on slowing cell cycle progression instead of any specific cell cycle arrest. In contrast, the cytotoxic effects include G2/M growth arrest, increased Bax/Bcl-2 ratio, mitochondria permeabilization, and apoptosis [314]. In lymphatic ECs (LEC) PCT inhibited their migration, adhesion, and branch point formation by activating autophagy mechanism independent of Akt phosphorylation. In vivo, the permeability of LEC to cancer cells was increased and this effect was ameliorated by the inhibition of autophagic flux. From the clinical point of view these effects of PCT were ambivalent as from one side it effectively reduced tumor size, it also promoted its spread to the sentinel lymph nodes [319]. HDMECs treated with PCT responded, in turn, with decreased proliferation and tube formation, and augmented necrosis [317].

DCT also exerts antiangiogenic effects, but their mechanism is slightly different. The drug inhibited HUVEC migration and tube formation, but not proliferation. It was associated with reducing the reorientation of the cell’s centrosome, acting as the microtubule organizing center [320]. In addition, the antimigratory activity of DCT was linked with ubiquitination and subsequent proteasomal degradation of HSP90, which blocks signals from the focal adhesions causing ECs’ incompetence to interact with VEGF. The drug also attenuated the VEGF-dependent phosphorylation of focal adhesion kinase, Akt, and eNOS, acting as downstream targets for HSP90 [321]. Another research on EA.hy926 hybrid endothelial cells showed that DCT inhibits their proliferation, migration, and invasion and induces apoptosis via the upregulation of p53. Moreover, it inhibited the adhesion of ECs by downregulating adhesion proteins VE-cadherin and PECAM-1. As per effectory signaling, DCT-related inhibition of EC migration and adhesion was controlled by VE-cadherin mediated integrin β1/FAK/ROCK transduction pathway [322].

Because EC senescence is involved in the pathogenesis of CVS disorders, such as atherosclerosis, hypertension, and thrombosis [83, 323, 324], the effect of taxanes on this phenomenon was tested. Research on HUVECs showed that PCT induces SA-β-Gal dose-dependently (2.5–5.0 nM), which coincided with increased prothrombotic PAI-1, one of the endothelial SASP indices [325]. At the same time, the level of eNOS decreased, imposing a vasoconstricting tendency. Although PCT-treated cells also displayed downregulated SIRT1, a mediator of senescence [326], the overexpression of this protein did not translate to ameliorated senescence EC phenotype [327]. The mechanism of PCT-induced HUVEC senescence also probably does not involve oxidative stress, as the MDA and GSH in treated cells remained unchanged [328]. However, this assumption should be clarified, as the experiments in vivo on PCT-treated rats revealed increased ROS release and NADPH oxidase mRNA in arteries and human coronary artery endothelial cells (HCAECs) [329].

As per the secretory prothrombotic profile of ECs, apart from increased PAI-1 [325], PCT potentiated tissue factor (TF) production and reduced expression of thrombomodulin and protein C receptor. Significantly, these effects were achievable only in the presence of a proinflammatory agent, TNFα, acting as the costimulator [330]. This finding does not discount the prothrombotic potential of PCT, as the drug is known to upregulate TNFα production by immune cells [331].

#### Vascular smooth muscle cells

A body of reports concentrated on PCT usage in drug-eluting stents (DESs) employed during PCIs in patients with coronary artery disease [332]. Because this procedure has some drawbacks, including mechanical damage leading to EC denudation and stimulation of both intravascular thrombosis and smooth muscle proliferation [333–336], PCT offers clear advantages because it restricts SMC expansion. The experimental background certifying antiproliferative PCT activity towards SMC is quite extensive. Human arterial smooth muscle cells (haSMCs) exposed to PCT displayed inhibited proliferation along with the stabilization of the microtubule network. The in vivo administration of the drug in rabbits led to vessel remodeling, manifested by reduced intima wall area, wall thickness, and magnitude of stenosis [337]. Time- and dose-dependency of SMC response to PCT clarified further experiments on human arterial cells showing that the drug arrests cell growth in the G2/M phase of the cell cycle, which was accompanied by their decreased ability to migrate [338]. A direct comparison of PCT activity against rat thoracic SMC and ECs showed that the antiproliferative reaction of the former was less dynamic, which was associated with a slightly different pattern of cell cycle regulation [339]. A dose-dependent reduction of SMC proliferation was also reported with respect to DCT, which exerted its action via downregulating cyclin-dependent kinase 2 (CDK2), CDK4, cyclin D1, cyclin E, retinoblastoma protein (pRb), and PCNA. The taxane also suppressed the phosphorylation of ERK1/2 and Akt [340]. The effectiveness of PCT delivery in restenosis prevention was demonstrated using various models, including humans, in whom the PCT-eluting stents provided considerable benefits in 6-month angiographic follow-up [341]. The positive outcomes of this drug administration were also found in rats, in which PCT inhibited pulmonary hypertension by modulating vascular remodeling, in particular, reducing the right ventricular hypertrophy index and thickness of the pulmonary arterial tunica media [342].

### Antimetabolites

Epidemiologic data indicate that 5-fluorouracil (5-FU) and its prodrug, capecitabine, are the second most common triggers of chemotherapy-related CVS disorders after anthracyclines [343, 344]. Cardiotoxicity was found in up to 35% of patients receiving 5-FU, and its extent differs depending on the dose and dosage algorithm [345]. Because of a relatively short half-life of 5-FU, which approximates 15–20 min [346], the cardiovascular consequences of the bolus drug administration appeared to be lower than those associated with its continuous intravenous delivery [347].

#### Cardiomyocytes

The mode of cardiotoxic activity of antimetabolites is wide and includes either indirect or direct toxicity. As per the first mechanism, leading to cardiomyopathy, 5-FU drives it through ischemic injury resulting from coronary vasospasm [348, 349]. As per the second mode, cardiomyocytes undergo direct damage via different pathomechanisms. In rats, 5-FU induced structural changes in the myocardium, including cytotoxicity (marked according to increased CK-MB and cTnI), hypertrophy, and dilatation. The damage in aged animals was more significant than in their young counterparts. Echocardiography showed that the drug causes ventricular enlargement and decreases myocardial contractile function. The left ventricular ejection fraction (LVEF) was also reduced, but this feature again only concerned the aged rats [350]. Similar findings derive from experiments on mice, in which left ventricular function indicators, such as ejection fraction, fractional shortening (FS), and cardiac output, were reduced upon treatment with 5-FU, whereas the levels of ventricular end-diastolic anterior wall thickness and LAID were increased [351]. Human cells exposed to 5-FU responded at decreased proliferation rate when the drug dose was 10 μM or higher. The exact concentration also appeared to be a threshold at which the first signs of apoptosis occur [352], staying in line with cardiomyocyte apoptosis in rats exposed to 5-FU in vivo [350]. Importantly, micromolar concentrations that effectively change cardiomyocyte functioning are in the range of therapeutic dosing of 5-FU and its prodrug, capecitabine [353–355]. The antiproliferative effects of 5-FU may be directly associated with blunting of CITED4, a key regulator for cardiomyocyte growth [356].

Another phenomenon elicited in cardiomyocytes by 5-FU is autophagy, albeit the concentrations capable of inducing this process are significantly greater (1 mM) than those with cytostatic or apoptotic activities. The extent of autophagy was inhibited by N-acetylcysteine, indicating that oxidative stress exacerbated in these cells leads to this kind of cell remodeling phenomenon [352].

Mechanistically, the toxicity of 5-FU and capecitabine is associated with oxidative stress. Human cardiomyocytes started overproducing ROS at as low concentrations of 5-FU as 0.01 μM [352]. Rat cardiomyocytes exposed to 5-FU and capecitabine displayed increased ROS production combined with depleted GSH and accumulated MDA [351, 355]. Myocardial tissue from guinea pigs exposed to 5-FU showed decreased SOD and GPX activities and increased CAT, which coincided with elevated MDA levels [357]. The intensified oxidative stress originated from mitochondria displaying decreased ΔΨ_m_ and transmitted to increased apoptosis and necrosis. A direct comparison of the two drugs showed that 5-FU is more cytotoxic than capecitabine [355]. Mitochondrial origin of 5-FU-induced ROS confirmed experiments on H9c2 rat cardiomyocytes, which when treated with this drug overproduced O_2_^−^ [358]. Also, the myocardium of rats treated with 5-FU in vivo experienced severe mitochondrial injury, manifested by reduced ΔΨ_m_ value and ATP level. These features were accompanied by morphological deterioration of these organelles in the form of swelling, partial fusion, and ridge breakage. Moreover, an increased number of autophagosomes was found, implying the induction of mitochondrial autophagy (mitophagy) [350].

Changes observed in mitochondria of 5-FU-treated cardiomyocytes included reduced size, crumpled membranes, and small cristae [351], indicating the activation of ferroptosis, the iron-dependent cell death [359]. The level of Fe^2+^ in the cardiac tissue was increased in response to the drug, and when the ferrostatin-1 (Fer-1), an inhibitor of ferroptosis [360], was used, MDA and GSH contents, initially elevated and decreased upon 5-FU exposure, respectively, changed in the opposite direction indicating the Fe-dependent kind of oxidative stress induction. In addition, Fer-1 relieved the diminished expression of glutathione peroxidase 4 (GPX4), the key inhibitor of ferroptosis [361], which suggests the causative involvement of this type of cell death in 5-FU-mediated cardiotoxicity [351]. The results of in vivo and in vitro studies further strengthened this statement by showing that 5-FU intensifies ferroptosis by reducing the expression of GPX4 and ferritin heavy chain 1 (FTH1) and elevating the expression of transferrin receptor 1 (TfR1) [362].

Energetic deficits in cardiomyocytes treated with 5-FU reported as a result of its direct activity, e.g., in guinea pig myocardium [363], may also proceed indirectly. Fluoroacetate, a catabolite of 5-FU, was found to inhibit aconitase, an enzyme engaged in the tricarboxylic acid cycle (TAC) progression and energy production [364]. Decreased ATP probably activates a vicious cycle in TAC because 5-FU appeared to stimulate α-KGDHC activity [365], the level of which is negatively correlated with ATP content [366]. At the same time, the redox disequilibrium elicited by 5-FU does not include the induction of anaerobic glycolysis, which was showed by experiments on H9c2 cells maintained under hypoxia [367].

An additional mechanism by which 5-FU impairs cardiomyocytes is the induction of cellular senescence, which occurs at concentrations as low as 0.1 μM [352]. Human cardiomyocytes exposed to 5-FU display increased mRNA for p16, a marker of premature cell senescence, and IL-6, exemplifying SASP [368].

#### Vascular endothelial cells

The development of myocardial ischemia in patients treated with 5-FU and capecitabine is causally linked with alterations within vascular endothelium [345, 369]. Scanning electron microscopy of rabbit vessels showed that the administration of 5-FU leads to severe ECs damage, including intima disruption and denudation of underlying structures, with accompanying platelet accumulation and fibrin deposition [370]. The drug used at 10 μM inhibits HUVEC proliferation and initiates apoptosis. At the same time, conversely to cardiomyocytes, only weak signs of autophagy were found, whereas the induction of senescence was evident between 10 and 1000 μM [352]. Hybrid endothelial cell line, EA.y926, subjected to 5-FU (100 μg/ml), displays reduced proliferation, enlarged and flattened morphology, as well as elevated SA-β-Gal, p16 and VCAM-1 mRNA [371]. Causatively, the induction of senescence was mediated by p38 MAPK and JNK, the inhibition of which prior 5-FU exposure alleviated the senescence phenotype. In addition, the drug decreased eNOS activity and SIRT-1 level in p38 MAPK-dependent mechanism. Interestingly, although 5-FU is known to upregulate ROS release in HUVECs and induce their senescence [352], in the case of EA.hy926 cells, the overproduction of ROS did not approach suggesting that senescence of these cells was uncoupled from oxidative stress [371]. Another exciting finding that had a translational nature was the induction of SA-β-Gal in cultured EA.hy926 cells in response to sera from patients treated with capecitabine. The signaling and secretory profile in these cells changed analogically as in cells exposed to 5-FU [371].

The toxic effect of 5-FU on ECs affecting eNOS-NO may result in coronary spasms and vasoconstriction [371]. Some role in these phenomena may also be played by endothelin-1 (ET-1), the level of which was increased in angina patients receiving 5-FU, likely due to its overproduction by ECs [372]. Increased aortic content of ET-1 was also recorded in Wistar rats treated with 5-FU, and it was accompanied by increased thromboxane A2, (TXA2), another vasoconstrictive agent [373], and reduced phosphorylation of eNOS [374]. In some cellular models, such as bovine ECs, deteriorated proliferation is compensated by a simultaneous elevation of anti-thrombotic factors, like prostacyclin [375].

#### Vascular smooth muscle cells

Coronary vasospasms resulting from 5-FU exposure are also related to altered functioning of vascular SMCs. Rings of aorta isolated from rabbits that were subjected to 5-FU in vitro displayed vasoconstriction independent from ECs, and this effect was controlled by PKC [376]. Fludarabine, a nucleoside analog, inhibits rat SMC proliferation in vivo and neointima formation after balloon angioplasty in vivo. These activities were regulated via the inhibition of STAT-1. This research, enriched in positive results of fludarabine-eluting stents application, provided evidence that besides causing cardiac ischemia, there are some benefits from antimetabolite usage, e.g., the reduction of in-stent restenosis [377]. Potential prevention of restenosis based on the antiproliferative effect of 5-FU and cytarabine was shown in studies on cultured porcine artery SMCs [378].

### Proteasome inhibitors

Proteostasis is one of the essential intracellular processes regulating the biogenesis, folding, trafficking, degradation, and turnover of proteins within a cell [379]. A protein cargo that is unfolded, misfolded, or no longer required to the cell is tagged by ubiquitin to be degraded by an ATP-dependent enzymatic system called a proteasome [380]. Malfunction proteostasis results in various pathological states, including CVS disorders [381]. Among relatively new generations of anticancer drugs are proteasome inhibitors. Some of them, especially carfilzomib (CFZ), exert cardiotoxic activity, including HF and cardiac arrhythmias [382]. The incidence of these adverse cardiovascular events in patients with multiple myeloma, to whom the proteasome inhibitors are clinically designated, reaches 3%–6% [383, 384].

#### Cardiomyocytes

Because of their minimal proliferative capacity, cardiomyocytes are exceptionally vulnerable to proteasome inhibition. The proteasome activity in cardiomyocytes is also higher compared with other tissues [385]. The suppression of proteasomal protein degradation and turnover leads to proteostasis disequilibrium manifested by an inappropriate accumulation of ubiquitinated proteins that gather, forming large, heterogeneous cytotoxic aggregates [386, 387]. The chymotrypsin-like proteasomal activity of CFZ was revealed in a preclinical pharmacokinetics study and linked with its potentially damaging activity toward mouse hearts at clinically relevant doses [388, 389]. Cardiotoxicity may also result from preventing mature myofibril formation in the cardiomyocytes [390].

Intracellular aggregation of polyubiquitinated proteins in cardiomyocytes triggers autophagy, evidenced in cells undergoing an experimental inhibition of the proteasome. The induction of autophagy is then considered the solution aimed at unloading the aggregate formation within the heart [391]. Molecular analysis performed on mice treated with CFZ (8 mg/kg) revealed that it causes moderate contractile dysfunction and left ventricular dilation, accompanied by the inhibition of AMP-activated protein kinase α (AMPKα) phosphorylation and inactivation of PI3K/Akt/eNOS signaling. Taking into account that AMPKα regulates autophagic degradation via suppression of mTOR complex 1 (mTORC1) [392, 393] and CFZ also downregulates Raptor phosphorylation that translates, in turn, to mTOR signaling inhibition [394], the drug appears to inhibit autophagy, which contradicts findings obtained in culture conditions [391]. At the same time, animals treated with bortezomib (BTZ) did not experience any functional cardiac injury nor altered AMPKα activation [388]. The last finding derived from in vivo experiments agrees with clinical observations pointing to a less cardiotoxic nature of BTZ compared with CFZ [395, 396]. It is also in keeping with the results of comparative analysis of CFZ, BTZ, and ixazomib (IXA), which showed that the most explicit activity on cardiomyocytes of the former is associated with its β5/β2-specific proteasome subunit inhibition pattern [397].

Another fate of cardiomyocytes (primary rat neonatal cells) exposed to CFZ and BTZ is apoptosis that proceeds time-dependently and results from attenuation of proteasomal-dependent constant sarcomeric protein turnover. Notably, when the cells were shortly preexposed to non-toxic doses of both proteosome inhibitors, their sensitivity to the damaging activity of DOX seriously increased, implying the additive mode of these drug cooperations [398].

Besides the direct impact of proteasome inhibitors on protein turnover and the consequences of its malfunction, there are other indirect effects of these drugs associated with alterations in general cellular metabolism and organelles, including mitochondria. These disturbances lead to energy deficits and defective contractility. As shown in hiPSC‐CMs exposed to CFZ (0.01–10 µM), the drug causes a dose- and time-dependent deterioration of cardiomyocyte viability combined with the augmentation of caspase 3/7 activity, suggestive of increased apoptosis. The mitochondrial origin of apoptosis confirmed diminished ΔΨ_m_ values, ATP production, mitochondrial oxidative respiration, and increased mitochondrial ROS. Altered mitochondrial metabolism coincided with decreased expression of transcripts engaged in the regulation of mitochondrial function, such as: *COQ10A* (coenzyme Q10A), *MFN1* (mitofusin 1), *MFN2* (mitofusin 2), and *NDUFB5* (NADH: ubiquinone oxidoreductase subunit B8). The cells displayed impaired contractility in 3D spheroids, which was also associated with compromised Ca^2+^ transients and decreased integrin‐mediated traction forces. The dysfunctional contractility was possibly caused by reduced expression of genes regulating calcium handling, including *SLC8A1* (solute carrier family 8 member A1), *RYR2, CASQ2* (calsequestrin 2), and *ATP2A2* (ATPase sarcoplasmic/endoplasmic reticulum Ca^2+^ transporting 2). In addition, CFZ decreased the expression of genes directly associated with cell contractions, that is *MYH6* (myosin heavy chain 6) and *MYL2* (myosin light chain 2) [399].

#### Vascular endothelial cells

Endothelial dysfunction participates in adverse cardiotoxic effects of proteasome inhibitors. Brachial artery flow-mediated dilatation allowed the demonstration that CFZ impairs endothelial function in a mechanism involving 26S proteasome activity inhibition [400]. Prothrombotic events fostering myocardial infarction, the significant negative consequence of CFZ activity [401], including thrombotic microangiopathy [402], may be related, in turn, to CFZ-dependent activation of complement, in particular, the deposition of membrane attack complex (C5b-9) on ECs [403]. Proteasome inhibitors may also indirectly exert their effects on ECs e.g., by modulating multiple myeloma cell secretome. BTZ promotes the production of extracellular vesicles (EVs) by the malignant cells, and these particles displayed decreased expression of several angiogenesis mediators, such as vascular endothelial growth factor (VEGF), interleukin 6 (IL-6), and basic fibroblast growth factor (bFGF). Moreover, these EVs suppressed the angiogenic activity of HUVECs, as evaluated using wound healing and tube formation assays. When the HUVECs were subjected to BTZ-generated EVs, they exhibited decreased NF-κB activation and attenuated secretion of autologous angiogenic agents [404]. These findings agree with the observation that BTZ also directly inhibits the proliferation of HUVECs, leading to increased apoptosis. At the level of cell cycle regulation, the drug prevented the G2/M transition and stimulated cyclin B1 and the cdc2/cyclin B complex. The effect of BTZ was likely associated with decreased secretion of proangiogenic VEGF, IL-6, insulin-like growth factor 1 (IGF1), and angiopoietin 1 (ANG1) [405]. Further research showed that the responsiveness of ECs to BTZ depends on their density. Namely, when the cells were confluent, they were resistant to the drug. However, the sub-confluent cultures reacted to BTZ in decreased angiogenic behavior. Intriguingly, the culture density also corresponded to the type of cell death elicited in those cells. In agreement with the previous report [405], sub-confluent cells displayed an increased incidence of apoptosis. In contrast, the confluent BTZ-treated cells initiated autophagy, which was preceded by increased production of ROS [406].

Research on isolated rabbit hearts and aorta also showed that CFZ affects vascular tone and reactivity, which agrees with clinical observations of its spasmogenic activity. CFZ was found to potentiate the vasospastic action elicited by KCl, noradrenaline, and angiotensin II, and impairs the vasodilation following the administration of nitroglycerin and nifedipine. Moreover, aortic strips preincubated with this drug displayed disturbed relaxation after the delivery of acetylcholine, which implies that the vasodilatory effects of CFZ proceed via an EC-dependent mechanism [407].

#### Vascular smooth muscle cells

Primary murine SMCs and human aortic SMCs treated with CFZ display hypocontraction and increased ROS formation [408]. The altered contractility of vascular SMCs exposed to CFZ may result from disturbed K^+^ channels altering the amplitude and gating (e.g., efficacy of inactivation) of delayed-rectifier K^+^ current in response to membrane depolarization [409]. Moreover, primary human pulmonary artery SMCs exposed to BTZ and marizomib (MRZ) displayed inhibited hypoxia-induced proliferation and increased apoptosis marked by cleaved poly (ADP-ribose) polymerase-1 (PARP1), one of the caspase’s substrates [410]. These changes could contribute to BTZ-dependent alleviation of hypoxia-induced pulmonary hypertension. This activity is consistent with the beneficial effect of BTZ on right ventricular hypertrophy and vascular remodeling in hypoxia-exposed and monocrotaline-injected rats, which was linked with inhibited NF-κB and the TGF-β/Smad signaling [411, 412]. Pulmonary artery SMCs exposed to BTZ display attenuated hypoxia-induced elevation of Ca^2+^ and proliferation, which coincided with the normalization of hypoxia-dependent increase in HIF-1α, bone morphogenetic protein 4 (BMP4), and the hypoxia-driven decline in peroxisome proliferator-activated receptor-γ (PPAR-γ) expression [413].

### Anti-HER2 agents

The guidelines of the ASCO (2017) indicate that treatment with trastuzumab (TZM) alone, or its administration following lower doses of anthracyclines (e.g., DOX < 250 mg/m^2^ or EPI < 600 mg/m^2^), puts patients with cancer at increased risk of cardiotoxicity [4]; mainly left ventricular systolic dysfunction (LVSD) and HF [414]. These recommendations have been strengthened by later guidelines by the European Society for Medical Oncology (ESMO, 2020) [4]. As per the mechanism of TZM’s anticancer activity, it is a humanized monoclonal antibody directed against human epidermal growth factor receptor 2 (HER2) [415].The incidence of adverse cardiac effects in patients with breast cancer treated with TZM differs among clinical trials and dosage regimens and ranges from 0.7% to 7.25% [416, 417]. Another study showed that the incidence of cardiomyopathy might affect up to 30% of individuals during or following HER2-targeted treatment [418].

#### Cardiomyocytes

TZM reduces in cardiomyocytes the activity of HER2/neuregulin 1 (NRG1) pathway and its downstream PI3K/Akt and MAPK/Erk1/2 signaling, which are critical for cell survival [419]. Another HER2 downstream molecule that TZM targets is transcription factor E2F-1, playing an essential role in the maintenance of cardiac function [420]. ChIP-sequencing showed that the abrogation of E2F-1 cardiac tissue leads to declined expression of several genes associated with CVS development, cell growth, cardiac hypertrophy, energy metabolism, and stress response [421]. Electron microscopy examination of mice heart cardiomyocytes showed several ultrastructural alterations caused by TZM and impaired left ventricular performance [422]. The morphological disturbances within cardiac myofibers included their disconnection, stretching, and diminished thickness, which could imply inhibition of their contractile activity. This assumption was confirmed in functional observations of TZM-treated animals, which displayed declined LVEF and FS. Transcriptomic analysis revealed that this impaired cardiac function, reflected by morphological and functional indices, has a solid molecular background associated with a downregulated expression of genes regulating contractility, stress response, and adaptation to hemodynamic pressure. These included: *My14*, *My17*, *Nppa*, *Ttn*, *Rxfp*, *Sln*, *Fgf12*, and *Fbx17* [422]. Another transcriptome sequencing report showed the downregulation of genes engaged in small molecule metabolism and cholesterol and sterol processing [423]. Specific changes also occurred within cardiomyocyte mitochondria, in particular, increased intermitochondrial distance and decreased the number of these organelles. The apparent mitochondrial dysfunction was plausibly the cause, and maybe also the secondary consequence, of increased oxidative stress, DNA damage, and apoptosis [422]. Induction of apoptosis, associated with a sharp elevation of proapoptotic Bcl-xS and declined antiapoptotic Bcl-xL, is indeed a fate of cells in which HER2 is inhibited, as shown on neonatal rat cardiomyocytes [424].

Mitochondrial dysfunction and altered cardiac energy metabolism were also recognized as the causes of disturbed contractile (decreased contraction velocity and deformed distance of monolayered cells) and Ca-handling (reduced amplitude and prolonged removal) properties of iPSC-CMs, but in contrast to the above-cited research, these effects occurred without increased ROS, apoptosis, or sarcomere disorganization [425]. A consistent view of cardiomyocyte mechanical properties also provided a study on mouse cardiomyocytes. ROS production and MDA level were increased in this case, accompanied by the loss of mitochondrial cristae, mitochondrial shrinkage, and sarcomere distortion. Mitochondrial damage was associated with decreased activity of peroxisome proliferator-activated receptor-γ coactivator 1-α (PGC1-α), a cofactor of mitochondria biogenesis [426], and mitochondrial uncoupling protein 2 (UCP2), engaged in oxidative phosphorylation management [427]. The ultimate fate of TZM-treated cardiomyocytes was apoptosis, which was also present in vivo in the animals exposed to this drug along with the increased concentration of oxidatively modified DNA and downregulated expression of DNA damage repair enzyme, 8-oxoguanine glycosylase (OGG1). The level of ferroptosis, marked by GPx4 and SLC7A11, decreased in animals exposed to TZM. However, when the ferroptosis was blocked using its specific inhibitor, the intervention alleviated TZM-related changes in cardiomyocyte mechanical and intracellular Ca^2+^ phenotype. Taking into account that empagliflozin, a sodium-glucose co-transporter 2 (SGLT2) inhibitor [428], efficiently attenuates TZM-induced lipid peroxidation in cardiomyocytes and their contractile defects, and that this activity was nullified by experimental ferroptosis induction, one may assume that this particular kind of cell death exerts rather negative outcomes towards cardiac tissue upon HER2 blockade [429].

As per the causative contribution of oxidative stress in TZM-mediated cardiotoxicity, the negative role of this process was evidenced in research employing curcumin, chrysin and thymoquinone, natural antioxidants, and mitochondria-protective agents [430–432]. These compounds were able to alleviate impaired mitochondrial metabolism and oxidative damage and reduce and recover TZM-induced cardiac toxicity in vivo and in vitro [433].

Another consequence of HER2 signaling dysregulation by TZM is the inhibition of autophagy, the process in which the recycling of damaged proteins improves the functioning and survival of cells subjected to certain stressful conditions, e.g., in cardiomyocytes undergoing pressure overload [434]. TZM induces the phosphorylation of HER1-Y845 and HER2-Y1248 and activates ERK1/2. These events lead to the initiation of the mTOR-Ulk1 pathway, which inhibits autophagosome formation, decreasing the levels of effectory proteins Atg 5–12, Atg 7, Atg 14, and Beclin 1. The antiautophagic activity represented by TZM was not shared by another humanized monoclonal antibody, pertuzumab [435], also known to increase the risk of HF in patients with HER2-positive cancer [436]. The issue of TZM-dependent changes in autophagy may be, however, a bit more complex than initially thought. Experiments conducted on iPSC‐CMs showed that the direction of this process might depend on the drug concentration. At a low dose of 1 μM, TZM suppressed the autophagy, whereas at higher doses of 10 μM a significant augmentation of this process was found, which is linked by the authors of this study with higher production of ROS upon 10 μM of TZM [437]. Further transcriptomic analysis of cells exposed to lower doses of TZM showed that it induces an inflammation-like state, as evidenced according to upregulated expression levels of *IL1β*, *TNFRSF8*, and *PTGS1,* oversecreted kallikrein 5 (KLK5) and 8 (KLK8), and increased production of KLK5/KLK8 substrate, PAR2. Moreover, TZM promoted the phosphorylation of ERK1/2 and JNK. Considering the role of these kinases in proinflammatory signals arising in response to PAR2 activation [438, 439], their contribution to the formation of the inflammatory milieu in TZM-treated cardiomyocytes is indisputable [437]. TZM-driven inflammatory conditions confirmed research on rabbits where the drug caused myocardium infiltration with lymphocytes and macrophages [440].

Last, but not least, the synergism between TZM and DOX, yielding cardiotoxicity in patients undergoing combination therapy, is associated with the fact that the HER2 inhibitor is capable of inhibiting, similarly to DOX [176], the activity of Top2B in cardiomyocytes. As a result, DDR is initiated and manifested by the accumulation of DNA double-strand breaks (γ-H2A.X foci) and increased phosphorylation of ataxia telangiectasia and Rad3-related protein (ATR) in cells subjected to TZM. When TZM and DOX were added to cardiomyocytes, the inhibitory effect on Top2B, proliferation, oxidative stress, and apoptosis were remarkably stronger than when the drugs acted separately. Finally, the unique observation was that DOX upregulated the level of HER2 and its downstream signaling in cardiomyocytes, thus sensitizing them to TZM [441]. DOX toxicity was also intensified in hPSC-CMs cardiomyocytes treated with another HER2 inhibitor, lapatinib. The etiology of this synergism was linked with the upregulation of inducible NOS (iNOS) and the overproduction of NO. These findings were confirmed in vivo when the delivery of an iNOS inhibitor in mice subjected to lapatinib and DOX ameliorated myocardial apoptosis and systolic dysfunction [442]. Similarly, as in the case of DOX, TZM was also found to aggravate cardiotoxicity caused by IR, which was evidenced in H9C2 cardiomyocytes. The cells undergoing concurrent TZM and IR displayed increased DNA damage, ROS release, and apoptosis, and this exacerbation of toxicity was associated with inhibited phosphorylation of Akt [49].

#### Vascular endothelial cells

Overexpression of HER2 in breast cancer cells is strongly associated with increased angiogenesis and VEGF expression. TZM, affecting HER2, was found to inhibit the expression of VEGF, thus limiting the supply of nutrients and oxygen to malignant tissue [443]. Apart from this purely anticancer activity of TZM, its action also deteriorates ECs functioning, which may participate in cardio- and vasotoxicity in cancer survivors. TZM interferes with HER2 dimerization, and EC contribution to this process is associated with the endothelial origin of the natural ligand, which is required for the dimerization, that is, NRG-1 [293]. Direct exposure of ECs to TZM resulted in their increased adhesion to fibronectin, which was accompanied by decreased angiogenic behavior marked by their proliferation and invasion. Moreover, the cells displayed a reduced ability to synthesize heparan sulfate proteoglycans, including perlecan [444]. Since arterial heparan sulfate negatively correlates with tissue cholesterol [445], the above findings may imply that TZM causes proatherosclerotic vascular wall remodeling. As per perlecan, which is a significant heparan sulfate in the basement membrane interacting with ECM proteins and growth factors (e.g., VEGF, FGF, PDGF) [446], its deficiency was found to affect endothelium-dependent relaxation, likely due to decreased eNOS mRNA and protein [447]. These findings may indicate that TZM may also reduce NO production by ECs. The bioavailability of this major vasorelaxant is increased through the activation of protein kinase B (PKB), which participates in HER2 and HER4 receptor dimerization [448]. TZM inhibits this dimerization and restricts the cardioprotective actions of NRG-1 [449], and the suppression of the eNOS-NO axis translates to vasodilator tone deregulation.

#### Vascular smooth muscle cells

Vascular disorders like atherosclerosis [450] and hypertension [451, 452] are reported CVS complications in patients with breast cancer treated with TZM or TZM combined with anthracyclines. At the same time, the impaired biology of vascular SMCs is undoubtedly a pathophysiological element contributing to the initiation and progression of these pathologies [128, 453]. Despite these indications, the literature lacks experimental data linking SMC exposure to TZM and the development of the above-mentioned abnormalities.

### Immune checkpoint inhibitors (ICIs)

Cancer therapy using ICIs represents an antibody-oriented targeted approach primarily focused on restoring the anticancer potency of T cells. ICIs are designed to block molecular targets engaged in T-cell activity suppression and immune tolerance, such as cytotoxic T-lymphocyte antigen 4 (CTLA-4) and programmed cell death 1 (PD-1) ligand 1 (PD-L1) system [454]. Despite enormous success in employing various ICIs in oncology, hyperstimulation of the immune system causes considerable toxicity toward normal cells, including those forming the CVS [455]. Moreover, ICIs are responsible for a wide range of non-inflammatory CVS abnormalities, such as Takotsubo-like syndrome, arrythmias, cardiomyopathy, arterial vascular disease, venous thromboembolism, pulmonary hypertension, myocardial infarction, and pericardial disease [15, 455]. Of the listed ICIs-driven perturbations, myocarditis is the most common [456]. The development of monoclonal antibodies against immune checkpoints was initiated by those counteracting CTLA-4 (Ipilimumab) [457]. Afterward, antibodies blocking PD-1 (e.g., Nivolumab, Pembrolizumab) and PD-L1 (e.g., Atezolizumab) were developed, tested, and approved, which was followed by the development of next generations of ICIs targeting other elements of immune checkpoint system, such as PD-L2, TIM-3, LAG-3, TIGIT, BTLA, and VISTA [458]. Notably, patients with cancer treated with a combination of two ICIs, which strategy is known to potentiate positive clinical outcomes in several kinds of tumors [459], display increased frequency and severity of cardiotoxicity compared with those subjected to a monotherapy [460].

#### Cardiomyocytes

It cannot be excluded that cardiomyocytes resemble cancer cells in utilizing CTLA-4 and PD-1/PD-L1 mechanisms to restrict T-cell-dependent hyperactivation of the immune reaction. If so, ICIs may relieve T-cell suppression leading to excessive CD8^+^ T-cell activity within the heart and cardiotoxicity. In fact, PD-1 and PD-L1 are expressed in cardiomyocytes and CTLA-4 and PD-1 deletion in an animal model results in autoimmune myocarditis [461–464]. Endomyocardial biopsy in patients subjected to ICIs revealed inflammatory infiltration of the heart muscle, mainly by CD8^+^ T cells, with a smaller admixture of CD68^+^ or CD163^+^ macrophages and CD4^+^ T cells [465–467]. This effect was accompanied by cardiomyocyte necrosis, myofibrillar degeneration, and sarcoplasmic tubular dilatation [467]. Necrotic cardiomyocytes are often positive for C4d, indicating the contribution of the activated complement pathway [466]. Cardiomyocytes cocultured with peripheral blood lymphocytes and exposed to ipilimumab or nivolumab confirmed exacerbated inflammatory response, as evidenced according to the stimulation of p65 NF-κB, MyD88, and Nlrp3 inflammasome, as well as increased production of IL-1β, IL-6, IL-8, and TGF-β1. The cell viability was markedly decreased. The effects exerted in response to anti-CTLA-4 antibodies were more pronounced than those associated with PD-1 blockade [468]. Cardiomyocyte apoptosis was seen in C57/BL6J mice treated with a combination of anti-PD-1 and anti-CTLA-4. RNA sequencing showed increased expression of genes coding for oxidative stress and decreased expression of heart-specific transcripts, such as mesencephalic astrocyte-derived neurotrophic factor and heat shock 70-kDa protein 5 [469]. Another study utilizing BALB/c mice revealed that ICIs-related myocarditis proceeds with increased cardiomyocyte autophagy [470].

#### Vascular endothelial cells

ECs are susceptible to PD-1/PD-L1 axis modulation, and PD-L1 is induced on ECs in response to proinflammatory stimuli [471]. Endothelial expression of PD-L1 within the myocardium in cardiac patients was increased compared with the control group, and this augmentation was more pronounced in those individuals who earlier experienced myocardial infarction. Significantly, the level of PD-L1 appeared to negatively correlate with LVEF and positively correlate with left ventricular end-diastolic volume [472]. Experiments on mice showed that during CD8 ^+^ T-cell-induced myocarditis, IFNγ produced by T cells was responsible for the upregulation of PD-L1 on ECs, and the inhibition of this cytokine worsened clinical picture of the disease. An antibody-based inhibition of PD-L1 converted transient myocarditis to lethal disease delivering evidence that endothelial PD-L1 is pivotal for control of immune-mediated cardiotoxicity [462]. Inhibition of CTLA-4 also promotes proinflammatory EC activation, as demonstrated according to increased expression of ICAM-1 in aortic cells. This outcome corresponded to the increased formation of atherosclerotic plaques, especially in the aortic arch of experimental animals [473].

#### Vascular smooth muscle cells

As mentioned earlier, ICIs may predispose an individual to atherosclerosis development. Preclinical studies on mice revealed that anti-CTLA-4 antibody administration decreases the content of SMCs within atherosclerotic lesions, which is suggestive of a reduced plaque stability [473]. Experiments on animals revealed, in turn, that PD-L1 is expressed in SMCs of the thickened intima in the graft coronary arteries, and the antibodies against PD-L1 promote the progression of graft arterial disease. The administration of anti-PD-L1 antibodies intensified the extent of inflammation, as estimated according to increased IFNγ and TNFα levels in cardiac allografts. Subsequent in vitro tests showed that PD-L1 expression in SMCs increased upon exposure to IFNγ levels, along with their proliferation [474].

### An opposite side of cardiooncology, or the effect of cardiologic drugs on cancer cells

Although cardiooncology is classically viewed as a discipline focused on CVS disorders in oncologic patients, it may also describe the effects of cardiologic drugs in patients with cancer and malignant cells. At the same time, this way of thinking about cardiooncology is not yet well-rooted in basic research, and its clinical significance has not yet been sufficiently recognized and appreciated.

#### Acetylsalicylic acid

Acetylsalicylic acid (AA, Aspirin) is an irreversible inhibitor of two isoforms of cyclooxygenase (COX), COX-1 and COX-2 [475]. COX-1 is an enzyme that produces thromboxane A2 (TXA2) in platelets, promoting their aggregation. COX-2 plays a vital role during inflammation, generating prostaglandin E2 (PGE2). AA is used in secondary prevention of cardiovascular events, e.g., myocardial infarction and ischemic stroke [476], reducing clot risk. There is evidence to suggest an impact of platelets on cancer cells. Namely, they can prevent cancer cells from being recognized by the immune system [477], promoting cell proliferation and facilitating migration [478]. Moreover, AA can induce apoptotic mechanisms by increasing *Bax* and decreasing *Bcl-2* gene expression [479]. Inflammatory processes play an essential role in carcinogenesis [480], and PGE2 intensifies tumor growth and angiogenesis [481]. On the other hand, AA inhibits the overexpression of the *COX-2* gene in colon cancer cells, which translates to reduced inflammation [479]. This effect may underlie the decreased risk of colorectal cancer in patients who received AA [482]. Regular use of this drug also reduced the risk of esophageal cancer, gastric cancer, breast cancer, and prostate cancer [483], as well as diminished mortality of patients with colorectal cancer [484].

#### Cardiac glycosides

Cardiac glycosides are used in the treatment of congestive HF and atrial fibrillation when the ventricular rate increases. The effect of glycosides is to increase cardiac output, reduce the heart rate, and slow the conduction of the atrioventricular node. Cardiac glycosides bind to sodium- and potassium-activated adenosine triphosphatase (Na + K + ATPase) and regulate cell membrane electrical potential. Increasing intracellular Ca^2+^ and Na^+^, cardiac glycosides can induce apoptosis in cancer cells [485, 486]. Earlier studies suggested cytotoxic and antiproliferative effects of cardiac glycosides in cells of breast cancer, renal cancer, lung cancer, prostate cancer, melanoma, leukemia, and neuroblastoma [487]. On the other hand, there is also a report demonstrating a higher risk of breast cancer in cardiac glycoside users, which may be associated with the estrogen-like activity of these drugs. In addition, an increase in all-cause mortality in the group that used cardiac glycosides was found. The difference between the results of earlier preclinical studies and clinical trials is possibly due to the use of too high concentrations of cardiac glycosides in experiments in vitro, which are impossible to be reached in the human organism in vivo [488].

#### Statins

Statins are a group of drugs used to reduce low-density lipoprotein cholesterol (LDL-C) levels in the primary and secondary prevention of atherosclerotic cardiovascular disease (ASCVD) [489]. These drugs are primarily used to treat hypercholesterolemia and hypertriglyceridemia and are one of the cornerstones in the treatment of cardiovascular diseases. The primary mechanism of action of statins is the early suppression of hepatic cholesterol production through competitive and reversible inhibition of 3-hydroxy-3-methylglutaryl-coenzyme A (HMG-CoA) reductase. By this mechanism, statins inhibit mevalonate biosynthesis from HMG-CoA. The consequence of this action is inhibiting the production of non-steroidal derivatives of mevalonate, such as farnesyl pyrophosphate and geranyl–geranyl phosphate. These isoprenoid compounds activate signaling proteins, especially G proteins (Rho, Rac, Ras), which allow proteins to attach to the cell membrane and interact with other proteins, causing disturbances in proliferation, migration, cytoskeleton function, and death [490]. Experimental studies have shown that statins inhibit the progression and induce apoptosis in breast cancer cells and esophageal adenocarcinoma cells [491, 492]. Clinical trials confirm the protective effect of statins by showing a reduced risk of gastric and esophageal cancers [493, 494]. They also appeared to reduce colorectal and breast cancer mortality [495, 496].

#### ACEIs/ARBs

Renin–angiotensin system (RAS) undergoes inhibition by angiotensin-converting enzyme inhibitors (ACE-Is) and angiotensin II receptor blockers (ARBs). ACE converts angiotensin I into angiotensin II, which is a vasoconstrictor. ARBs blocking AT1 receptors inhibit angiotensin II. Both groups of drugs suppress the RAS, regulating vascular function and blood volume, which leads to decreased blood pressure. Experimental data showed local expression components of the RAS in brain, lung, breast, prostate, and pancreatic cancer cells [497]. A metaanalysis of 55 studies showed a significant relationship between RAS inhibition and patients with cancer overall survival, progression-free survival, and disease-free survival [498]. Sub-analysis, according to the RAS inhibitory group, showed that the effects of ARBs are considerably more robust than those of ACEI [498].

#### Beta blockers

Beta-adrenergic receptor blockers are a heterogeneous group of drugs that inhibit the excessive activity of the sympathetic nervous system, recommended for the treatment of arrhythmias, coronary heart disease, HF, and hypertension. Experimental studies showed that increased sympathetic nervous system activity may promote cancer development [499]. Conversely, metaanalyses of clinical trials showed no conclusive results about using beta-adrenergic receptor blockers in patients with cancer. Overall survival, all-cause mortality, disease-free survival, progression-free survival, and recurrence-free survival did not differ significantly among the beta-adrenergic receptor blockers group [500]. Nonetheless, in some types of cancers, including ovarian cancer, pancreatic cancer, and melanoma, an improvement in overall survival was found in the beta-adrenergic receptor blockers group [500].

## Conclusions and future directions

Generally speaking, the roots of cardiooncology come from clinical observations, and the same source determines the decision-making process regarding therapeutic management of oncology patients who developed cardiovascular complications. At the same time, knowledge of the pathomechanisms of these disorders derives from basic research on cellular and animal models. As this paper shows, CVS cellular and molecular mechanisms dysfunctions are well understood and explored down to the level of mediators and signaling pathways. On the other hand, specific issues still need to be clarified, especially where observations of the outcomes of particular drugs have been inconclusive. An excellent way to get meaningful findings will be to optimize research methods. For example, it seems desirable to increase the share of studies using animal organisms, tissue isolates, 3D cultures, and even cocultures, which have a significant advantage over isolated cellular models. The other aspect is the diversity of cell types engaged in therapy-induced cardiotoxicity. In this review, we arbitrarily selected three major types of cells. At the same time, we skipped discussing the role of other types, such as cardiac progenitor cells and fibroblasts, mesenchymal stem cells, and bone marrow cells, whose dysfunction may also be caused by radiation or drugs, and thus may also affect the functioning of the entire CVS. Last, but not least, much more attention must be paid to clinically relevant doses of IR and therapeutics utilized in vitro and in vivo, as the cellular and animal reactions to those stressors discussed in this article undoubtedly depend on their doses and/or times of exposure.

## Data Availability

Not applicable.

## Abbreviations

5-FU: 5-Fluorouracil
AA: Acetylsalicylic acid
ACT: Anthracycline‐induced cardiotoxicity
ACE-Is: Angiotensin-converting enzyme inhibitors
ARBs: Angiotensin II receptor blockers
BAEC: Bovine aortic endothelial cells
BTZ: Bortezomib
CFZ: Carfilzomib
CIS: Cisplatin
CPT: Carboplatin
CTLA-4: Cytotoxic T lymphocyte-associated protein-4
CVD: Cardiovascular disease
CVS: Cardiovascular system
DAMPs: Damage-associated molecular patterns
DCT: Docetaxel
DDR: DNA damage response
DOX: Doxorubicin
HDMECs: Dermal microvascular endothelial cells
ECs: Endothelial cells
eNOS: Endothelial nitric oxide synthase
EPI: 4′-Epidoxorubicin
hiPSC-CMs: Human induced pluripotent stem cell-derived cardiomyocytes
HF: Heart failure
HCAEC: Human coronary artery endothelial cells
HER2: Human epidermal growth factor receptor 2
HMEC: Human microvascular endothelial cells
HUVECs: Human umbilical vein endothelial cells
ICIs: Immune checkpoint inhibitors
IR: Ionizing radiation
LVDP: Left ventricular developed pressure
LVEF: Left ventricular ejection fraction
LVSD: Left ventricular systolic dysfunction
mPTP: Mitochondrial permeability transition pores
NCX: Na^+^/Ca^2+^ exchanger
NO: Nitic oxide
NOX: NADPH oxidase
PCIs: Percutaneous coronary interventions
PCT: Paclitaxel
PD-1: Programmed cell death protein-1
PD-L1: Programmed cell death 1 ligand-1
RAS: Renin–angiotensin system
RIHD: Radiation-induced heart disease
ROS: Reactive oxygen species
RyR: Ryanodine receptor
SA-β-Gal: Senescence-associated β-galactosidase
SASP: Senescence-associated secretory phenotype
Top2B: Topoisomerase IIB
TZM: Trastuzumab
VSMCs: Vascular smooth muscle cells
